# How the heterogeneity of the severely injured brain affects hybrid diffuse optical signals: case examples and guidelines

**DOI:** 10.1117/1.NPh.11.4.045005

**Published:** 2024-10-18

**Authors:** Susanna Tagliabue, Michał Kacprzak, Anna Rey-Perez, Jacinto Baena, Marilyn Riveiro, Federica Maruccia, Jonas B. Fischer, Maria A. Poca, Turgut Durduran

**Affiliations:** a ICFO–Institut de Ciències Fotòniques, The Barcelona Institute of Science and Technology, Biomedical Optics, Barcelona, Spain; b Nalecz Institute of Biocybernetics and Biomedical Engineering, Warsaw, Poland; c Vall d’Hebron Hospital, Neurotrauma Intensive Care Unit, Barcelona, Spain; d Vall d’Hebron Research Institute (VHIR), Neurotraumatology and Neurosurgery Research Unit (UNINN), Barcelona, Spain; e Vall d’Hebron Hospital, Department of Neurosurgery, Barcelona, Spain; f Universitat Autònoma de Barcelona, Barcelona, Spain; g Institució Catalana de Recerca i Estudis Avançats (ICREA), Barcelona, Spain

**Keywords:** hybrid diffuse optics, neurophotonics, multimodal neuromonitoring, atypical tissue effect, structural heterogeneities, data quality control, measurement guidelines

## Abstract

**Significance:**

A shortcoming of the routine clinical use of diffuse optics (DO) in the injured head has been that the results from commercial near-infrared spectroscopy-based devices are not reproducible, often give physiologically invalid values, and differ among systems. Besides the limitations due to the physics of continuous-wave light sources, one culprit is the head heterogeneity and the underlying morphological and functional abnormalities of the probed tissue.

**Aim:**

The aim is to investigate the effect that different tissue alterations in the damaged head have on DO signals and provide guidelines to avoid data misinterpretation.

**Approach:**

DO measurements and computed tomography scans were acquired on brain-injured patients. The relationship between the signals and the underlying tissue types was classified on a case-by-case basis.

**Results:**

Examples and suggestions to establish quality control routines were provided. The findings suggested guidelines for carrying out DO measurements and speculations toward improved devices.

**Conclusions:**

We advocate for the standardization of the DO measurements to secure a role for DO in neurocritical care. We suggest that blind measurements are unacceptably problematic due to confounding effects and care using *a priori* and *a posteriori* quality control routines that go beyond an assessment of the signal-to-noise ratio that is typically utilized.

## 1 Introduction

Both morphology (depth, location, and shape) and the wavelength-dependent optical properties of the underlying tissue affect the observed signals of diffuse optics (DO).^1^^,^^2^ These are often modeled to be homogeneous or as simplified layered signals when fitting them with a theoretical model to obtain the quantities of interest.^3^^–^^8^ This is done not because the researchers do not appreciate the complex relationship between the two, but rather because the details of that relationship are too complex to know and utilize for practical measurements. Nevertheless, some more advanced methods utilizing a large number of source–detector pairs to reconstruct tomographic images use the underlying tissue morphology derived from brain scans as *a priori* information.^9^^–^^11^ This comes at the cost of being less practical.

The availability of a complex model is not the only issue. Some of the atypical, heterogeneous tissues reduce the signal-to-noise ratio (SNR) to levels that would require unpractical solutions with the current technologies to obtain a reliable number of photons for data processing. This consequence is not a surprise because the physics of the photon propagation is well understood,^3^^,^^5^^,^^12^^,^^13^ and it is important to note that these heterogeneities also affect other modalities.

The lack of a universal agreement over the analysis methods and a reliable assessment of the limitations of the technology led to the failures of several clinical trials^14^^–^^17^ using commercial near-infrared spectroscopy (NIRS) oximeters for neuro-monitoring. This has been particularly impactful in the neurocritical care of conditions such as traumatic brain injury (TBI) and led the clinical community to debate over the utility of NIRS due to the observed limitations in reproducibility, reliability, and accuracy which has been reflected in both the literature^18^^–^^22^ and elsewhere (e.g., in various discussions in congresses and forums). Many early adopters were discouraged by these results and abandoned NIRS-based cerebral oximetry. It can be argued that this could have been addressed better by the manufacturers through a careful study of the relationship between the tissue morphology and the optical signals and with more advanced online quality control measures. As expected, similar problems arose in studies emerging from research groups that are the developers of the technology and were addressed on an *ad hoc* basis during the data analysis, mainly by discarding unreliable data.^18^^–^^22^ Detailed studies of this relationship were also carried out using simulations and with limited *in vivo* data.^23^^–^^32^

In this article, an analysis of the data obtained by time-resolved spectroscopy (TRS)^33^ and diffuse correlation spectroscopy (DCS)^3^ over several years using the patient brain scans as the guiding data is reported. We opted not to use this information to build a complex model (would have been unstable due to the limited number of measurements) but rather to use them as a set of case studies. It will be argued here that this is an important contributor to a controversial issue in the field. Our goal is to provide anecdotal examples and speculate on different actions that could be taken to minimize the impact of these complexities on both the clinical science and also the clinical trial levels.

From our presentation, it will be evident that this population is very heterogeneous, and each case should be inspected with respect to the quality of the optical signals as well as the potential ability to probe the brain through overlaying, complex tissue structures. The open question is whether these instructions or suggestions on how to improve the measurements and to develop indicators as to when to trust the results and/or to discard them could lead to a more successful future for near-infrared spectroscopic methods. Our goal is to offer an illustrated guide to assist end-users—such as developers of emerging commercial technologies, young researchers, and clinical collaborators—in applying diffuse optical methods for neuromonitoring in patients with critical care pathologies.

## 2 Materials and Methods

### 2.1 Diffuse Optical Instrumentation

Two similar hybrid DO devices were used in this study, both previously described in Refs. 34–36. Both devices are based on NIRS principles, and they combine TRS and DCS. TRS allows the retrieval of the wavelength-dependent absorption coefficient ($\mu_{a}$) and the reduced scattering coefficient ($\mu_{s^{\prime }}$) to derive the microvascular oxy- and deoxyhemoglobin concentrations and the tissue/blood oxygen saturation (${\mathrm{StO}}_{2}$).^3^^,^^33^ TRS is based on the measurement of the distribution of the time-of-flight (DTOF) of photons, which allows for the accurate separation of the absorption and scattering effects at each wavelength.^33^ TRS data were acquired every 1 s with a 3- to 5-mW light injection at 687 and 830 nm.

DCS uses a 785-nm continuous wave (CW) and coherent light source (27.5 mW) to quantify the statistics of the diffuse speckles to derive a blood-flow index (BFI) that is well known to be proportional to absolute cerebral blood flow.^37^

During the data acquisition, the optical probes were maintained fixed to the skin by self-adherent elastic bandages, i.e., kinesiology tape (Classic, Kintex, Talheim, Germany) and Coban wrap (Coban™, 3M Science Applied to Life, St. Paul, Minnesota, United States). Hand-held measurements without bandages were performed whenever the condition of the subject did not allow for the use of fixed probes, for example, due to the presence of decompressive craniectomy.

### 2.2 Brain Scans

Non-contrast 3D X-ray computed tomography (CT) scans of the head by a standard CT scanner (SOMATOM Definition AS or AS+, SIEMENS Healthcare, Erlangen, Germany) were performed by the expert personnel of Vall D’Hebron University Hospital (VHUH) whenever required for routine diagnosis. The available CT scans closest in time to the DO acquisitions were then used for the purposes of this study.

CT scans were performed with a maximum voltage ($V_{\max}$) of 120 kV, a current of 35 mA, and either 1- or 4-mm slice thickness (meaning the distance between a scanned plane and the subsequent). When present, the 1-mm slice resolution was always preferred to the 4-mm one for the following evaluation.

The acquired CT dataset for each instance was reconstructed with the hospital’s proprietary software, anonymized, and exported as a Digital Imaging and Communications in Medicine (DICOM) dataset for analysis using third-party software. A standard viewing software (RadiAnt DICOM Viewer, trial version 2021.1, Medixant, Poznań, Poland) was utilized outside the hospital to display DICOM data and with default settings for conventional CT images. Further information was removed using another user interface free tool (DicomCleaner™, PixelMed™ Toolkit).

### 2.3 Subject Inclusion Criteria and Study Protocols

Three different groups of patients present in the intensive care unit (ICU) of the VHUH were selected for enrollment in this study: a cohort of malignant middle cerebral artery (MCA) infarction patients, a cohort of subjects with different severity of TBI and with subarachnoid hemorrhage (SAH).

All cohorts obtained clearance from the local ethical committee within VHUH (ACU-AT-203/2012-3531) and conducted according to the Declaration of Helsinki.^38^ Informed consent was collected prior to the measurements and signed by either the patients or their legal representative.

In the case of the malignant MCA infarction cohort, patients with large hemispheric infarction were recruited for a prospective interventional study. The inclusion criteria were (1) the presence of malignant MCA infarction and a hypodense lesion involving at least 50% of the middle cerebral artery territory with or without decompressive craniotomy and (2) age between 18 and 65 years.

For the SAH cohort, patients were included in case of ischemic lesions related to subarachnoid hemorrhage and/or conditions following SAH [i.e., aneurysmal rupture with low Glasgow Coma Scale (GCS) score, bleeding, vasospasm, mild ischemia, and ischemic infarction]. These patients, aged more than 18 years, were included in a prospective interventional study.

In the TBI cohort, the inclusion criteria were the same as reported in Ref. 34. In summary, patients over 16 years old with a TBI, GCS score of 3 to 15 upon admission, who required ICP monitoring, and mechanical ventilation from onset or after clinical decline were enrolled. The eligibility criteria included normocapnia [partial pressure of arterial carbon dioxide (${\mathrm{PaCO}}_{2}$) of 35 to 40 mmHg] and stable hemodynamics allowing for a short hypocapnic challenge, with informed consent from a legal representative. The exclusion criteria were patients with contraindications for invasive neuromonitoring (e.g., anticoagulant use, scalp infection, and bleeding disorders) and clinical signs of brain death.

**Fig. 1:**
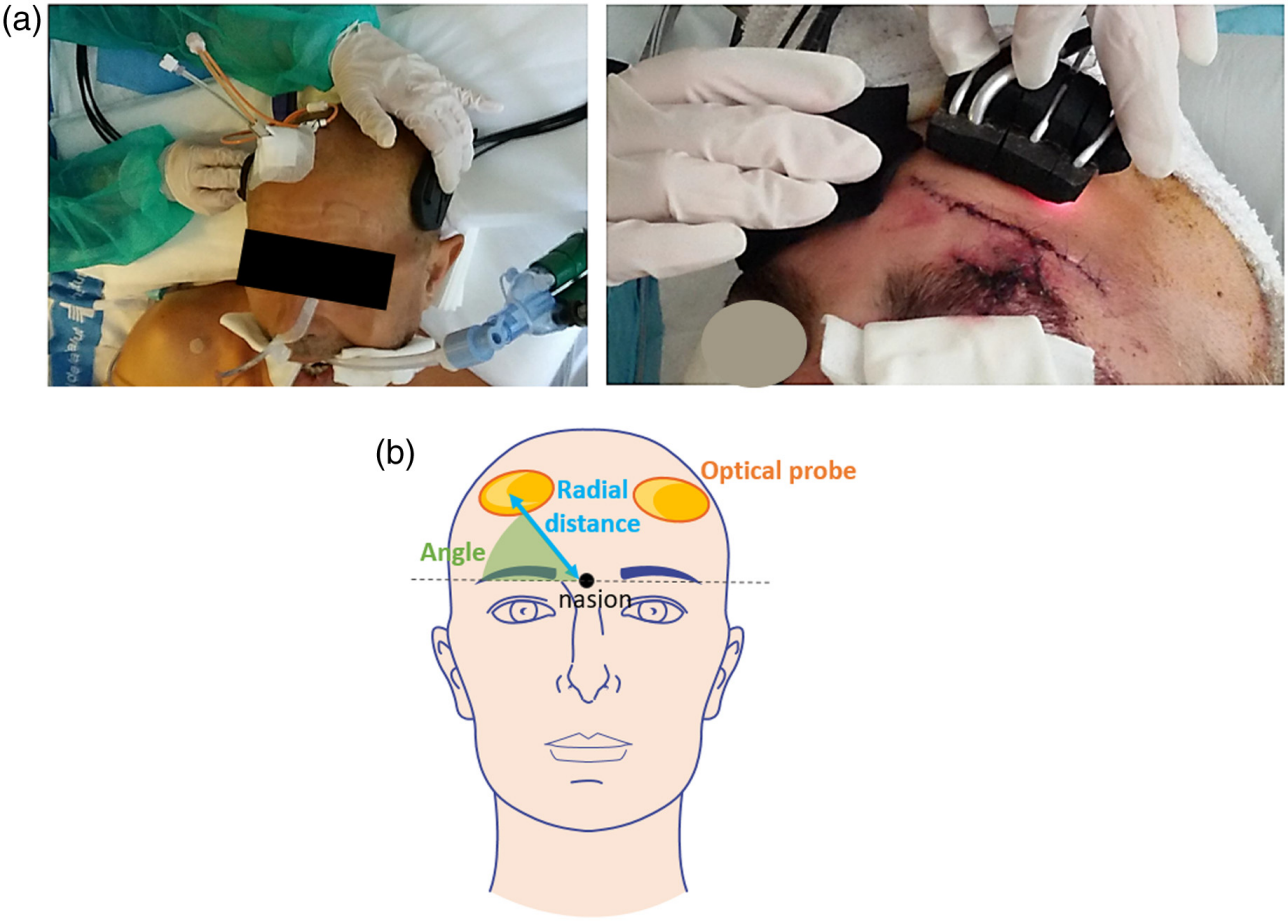
(a) Pictures during hand-held measurements. (b) Illustration of the placement of the optical probes and the recording of the placement in terms of angle and radial distance. The radial distance is measured from the nasion to the mark where the TRS bundle tip was leaning on. The angle was calculated considering the line that passes through the nasion and the eyebrows, perpendicular to the line of the nose, and the line used to calculate the radial distance (this figure was created by modifying images from Servier Medical Art,^39^ part of Laboratoires Servier, licensed under a Creative Commons Attribution 3.0 Unported License).

Despite the populations being different, the study protocol has been uniform: it consisted of ∼5  min of steady-state acquisition on selected positions on the head (Fig. 1).

The choice of the position for the measurements was made in collaboration with the expert medical staff who identified the different areas of interest according to the CT scans available prior to each measurement. Up to four positions were chosen on the same subject, and few subjects were measured multiple times during different acquisition sessions.

**Fig. 2:**
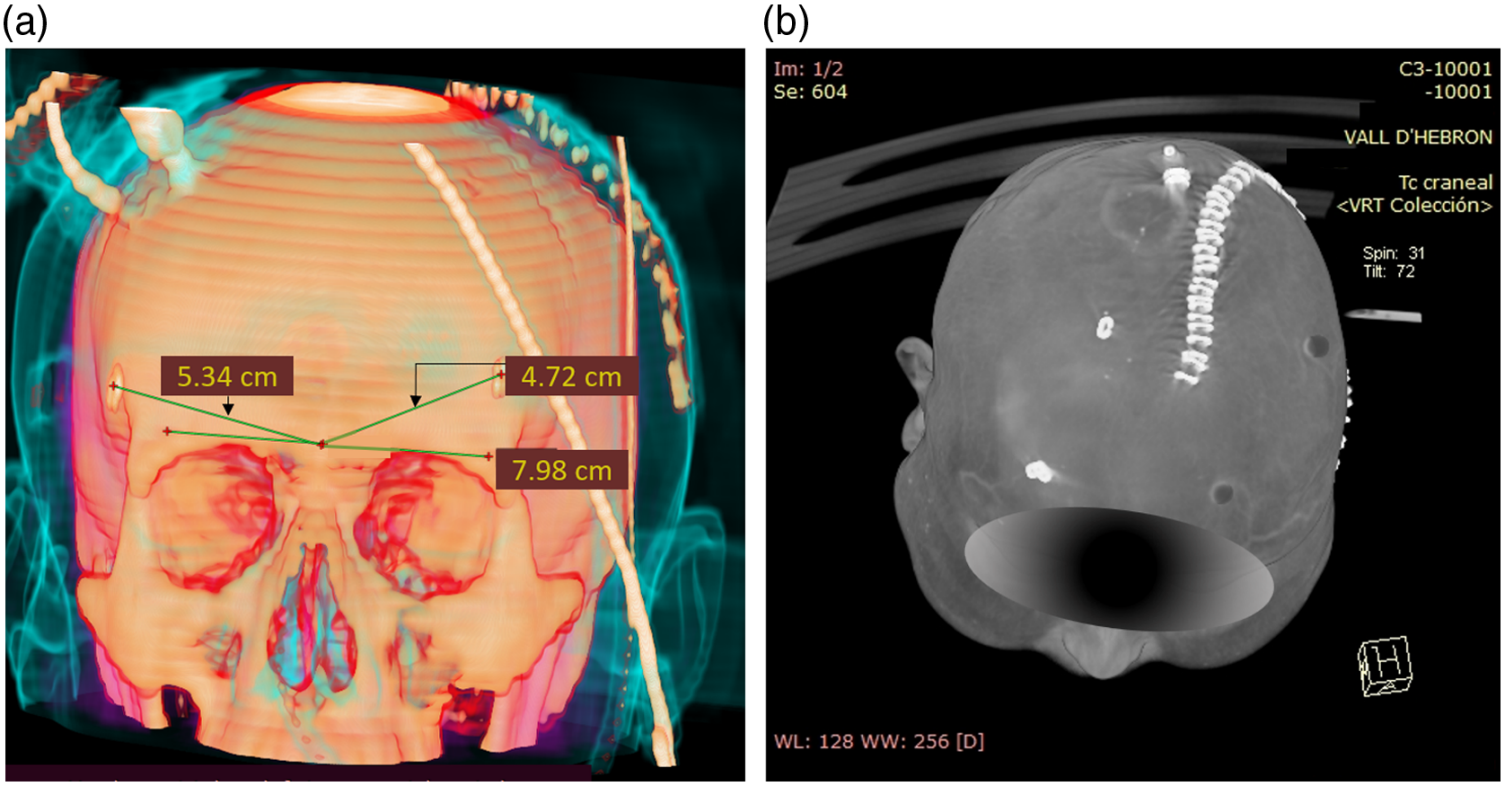
Examples of three-dimensional reconstruction of CT scans with fiducial references of the probes’ placements for two different subjects and measurements. (a) The distances calculated according to the procedure explained in the text are shown. However, it should be considered that these distances are actually shorter than reality, due to the fact that the software did not provide a way to follow the curvature on the skin, whereas it calculates the straight distance between points. (b) The very first trial for recording the positioning of the probes is presented. Two positions were marked by staples, whereas D vitamin capsules were used in the other two. Higher contrast was found for the staples, without producing huge artifacts on tissue and were therefore chosen for the study.

After the acquisition, the position of the probes on the head was recorded. First, the location of the TRS detection fiber tip was marked with a sterile surgical staple, which was fixed on the intact skin by standard transparent medical tape. Subsequently, the radial distance between the nasion and the staple was measured, as well as the angle spanning between this radial distance and the straight line passing through the nasion and the eye line, as visible in Fig. 2a. It was decided to use the TRS tip to represent the whole probe for convenience. To measure the radial distance and the angle, a flexible measuring tape and a protractor were used. Figure 1(b) shows an illustration to better understand these parameters. In cases where CT scans were taken after measurement, the staple position was then captured and visible in CT scans for post-processing reference, as shown in Fig. 2.

Additional demographic and clinical information such as presence of injury, presence of decompressive craniectomy, sedation state, presence of hematoma, gender, presence of bone, and age were also collected.

### 2.4 Data Evaluation

The optical data were analyzed following the physical models and procedures described in Refs. 13, 36, 40, and 41 by making use of MATLAB (Release 2018b, MathWorks, Natick, Massachusetts, United States). Once the optical data were fitted by the appropriate model, $\mu_{a}$, $\mu_{s}^{\prime }$ at both 687 and 830 nm were estimated by TRS, and ${\mathrm{StO}}_{2}$ was derived. Similarly, from DCS, a BFI was derived.

For the purposes of this work, a “baseline,” i.e., a period of constant hemodynamics, was assumed, and the mean values were calculated for all the variables at each measurement position on the head.

The CT scans were utilized in a more qualitative fashion to define regions of interest (ROIs) in correspondence with each probe position as detailed in Appendix A. We chose to consider an ROI as a circular area of 15-mm radius ($∼700\;{\mathrm{mm}}^{2}$) because the interrogated region below the probe is dominated by photons that have reached approximately this depth due to the interfiber distances.

**Fig. 3:**
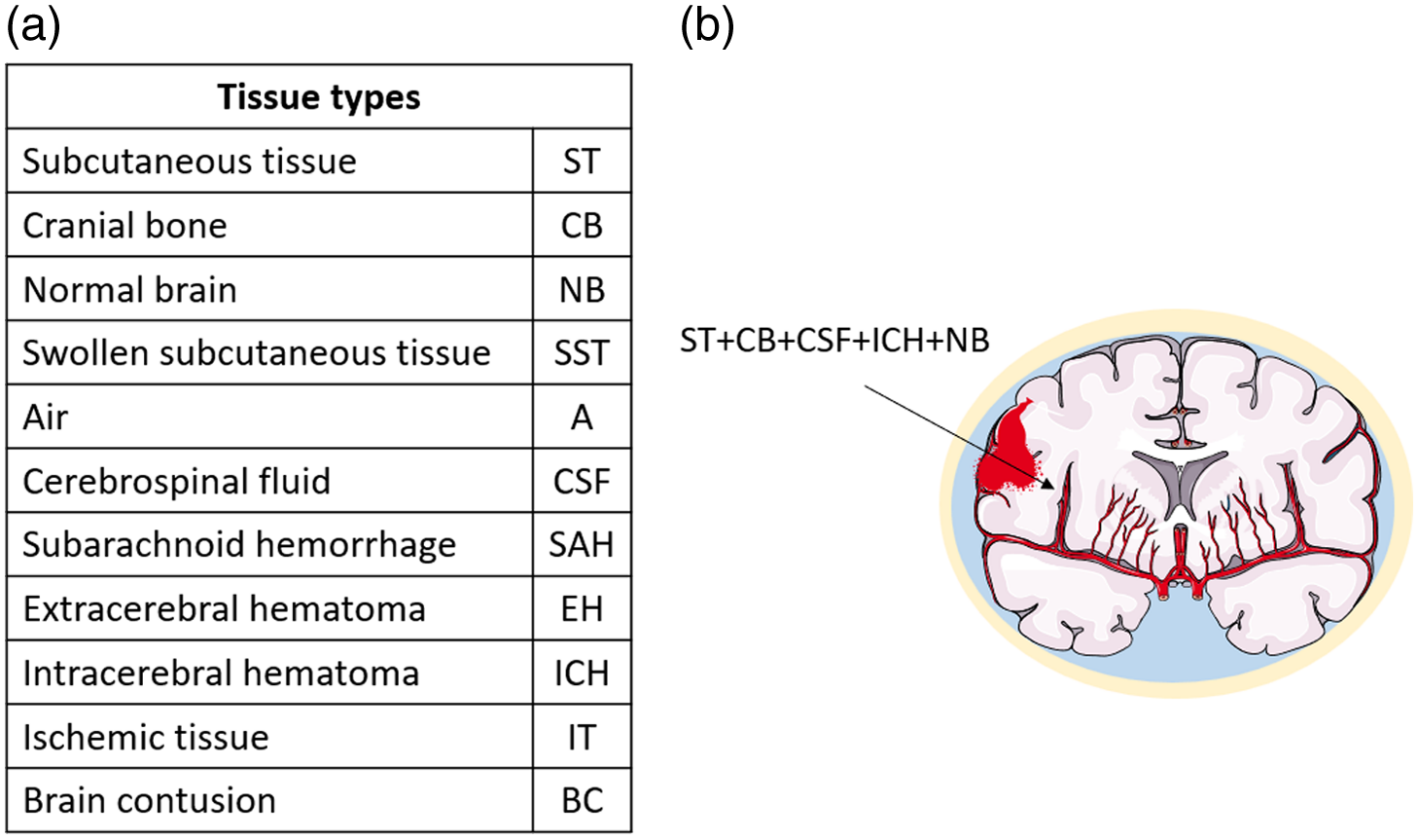
(a) List of tissue types for the qualitative description of the regions of interest below the optical probe position. (b) Example of tissue composition where a hemorrhage is present (this figure was created by modifying images from Servier Medical Art,^39^ part of Laboratoires Servier, licensed under a Creative Commons Attribution 3.0 Unported License).

As for the qualitative evaluation, the tissue composition below the probe was assessed by experienced clinicians and categorized. The following tissue types were selected and identified with an acronym: subcutaneous tissue (ST), cranial bone (CB), normal brain (NB), swollen subcutaneous tissue (SST), air (A), cerebrospinal fluid (CSF), SAH, extracerebral hematoma (EH), intracerebral hematoma (ICH), ischemic tissue (IT), and brain contusion. The combination of all tissue types going from the most external to the most internal in the head was listed per each ROI, providing a composition of different tissue types. The basic categories that were utilized are summarized in Fig. 3, and one example is provided. The skin was disregarded because it was common to all measurements. In Appendix B, we provide an example of swollen subcutaneous tissue compared with a regular one.

A database was created gathering all the measured variables from optical data, CT scans, clinical information, and the different categories of tissue properties.

### 2.5 Dataset Description

The created comprehensive dataset comprises three key components:

• patient data spreadsheet in .csv format (Microsoft Office Professional Plus 2021), compatible with Microsoft Excel and other spreadsheet programs, storing demographic and clinical information, optical data results, and tissue descriptions
• MATLAB data files in .mat format (Release 2018b, MathWorks, Natick, Massachusetts, United States) containing a structure that stores the acquired raw data and analyzed optical results gathered in a compressed file.zip
• CT scan images in DICOM format gathered in a compressed file.zip.

For the data spreadsheet, each row corresponds to a different measurement position, an ROI. Therefore, multiple measurements may be available for the same subject either for different optical probe placements within the same acquisition or for repeated acquisitions within the same measurement day and or on a different measurement day. The columns, instead, contain the measurement identification numbers, averaged values of the optical parameters ($\mu_{a}$, $\mu_{s}^{\prime }$, ${\mathrm{StO}}_{2}$, and BFI), and demographic and clinical information (tissue composition, presence of injury, presence of decompressive craniectomy, sedation state, presence of hematoma, gender, presence of bone, and age). Whenever the patient repeatedly underwent data acquisition, although the ROI was different, both clinical and demographic information were repeated for all data acquisitions. The “wavelength” column distinguishes optical properties obtained from the same probed region.

Regarding the MATLAB data files (namely, a structure array is a data type that groups related data using data containers called fields), they house a structured data format containing the raw and processed data, such as both optical coefficients calculated at each wavelength, the BFI, and the ${\mathrm{StO}}_{2}$ values at each ROI (maximum two ROIs per file). The structure also contains additional information for purposes beyond this research. Importantly, data acquired during the same measurement acquisition but with two distinct optical probe placements are stored separately within the structure: field “hemi1” contains the results for the first position and field “hemi2” for the second position. If multiple measurement acquisitions occurred on the same day or across different days, either for one or both of the optical probes, these are saved as a separate MATLAB file. In this case, all the generated data files are stored in separate folders with incremental measurement numbers.

CT scan images are provided for each measurement as described in Sec. 2.2. In cases where data acquisition occurred on the same day (or closely enough in time), a single CT scan may be used for multiple ROIs.

### 2.6 Data Analysis

The bulk of the results are demonstrated as case examples by investigating the measured optical signals and their detailed structure to compare them with the clinical and radiological findings. Moreover, we used the results from a healthy volunteer to provide a fairly representative scenario for comparison against the data from pathological or abnormal cases.

**Fig. 4:**
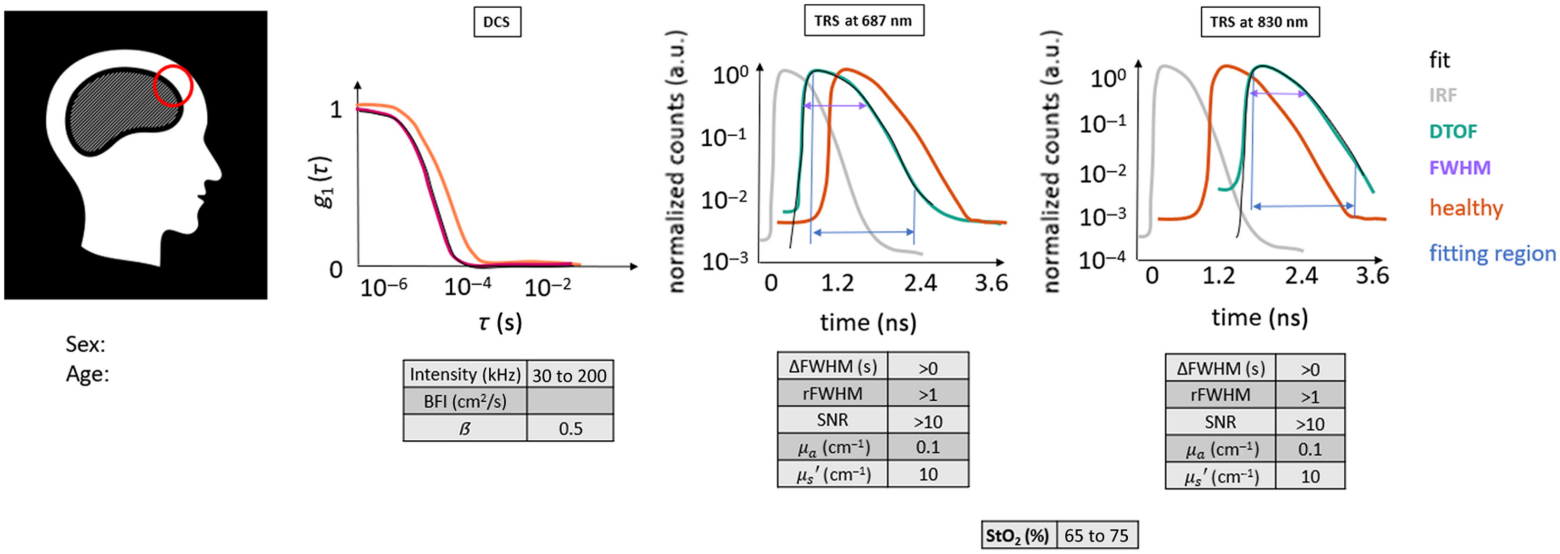
Scheme that allows one to appreciate the salient figures of the curves related to the DCS and TRS methods and to relate their shape with the characteristics of the tissue under scrutiny of the optical probe. The position of the latter is shown on the CT scan by a red circle. Furthermore, indicative ranges and values for the tissue of a healthy adult human head are provided in tables for the different modalities. This scheme will be the same one used to elucidate the clinical cases chosen in the following section. Red circle: ROI (Ø30  mm). Magenta curve: current subject’s $g_{1}(\tau )$ curve.

Each case example is elucidated by the same set of information, whenever possible, as exemplified by Fig. 4. Both optical reconstructions and essential evaluated parameters are also reported as descriptors of the probed region.

From the left, the corresponding slice of the CT scan of the patient is shown with the marked ROI on it. Below, the age and sex of the subject are reported.

Then, DCS results are presented as the normalized electric field autocorrelation function [$g_{1}(\tau )$, with τ as the delay time] derived from the normalized intensity autocorrelation function.^3^ For each probe position, $g_{1}(\tau )$ curves were averaged over a hundred consecutive as well as over the different detection channels. Therefore, only one autocorrelation curve is represented for the selected ROI, as in Fig. 4. Afterward, the fitting procedure was performed on this curve to retrieve BFI. The obtained fit is superimposed to the averaged $g_{1}(\tau )$ curve in this figure to appreciate how much the data are represented by the ideal model. In the same graph, the $g_{1}(\tau )$ curve obtained in the same way from the healthy volunteer is plotted in orange as a reference. In addition to $g_{1}(\tau )$, for each averaged curve, we have reported the information about the instrument β parameter, count rate (intensity), and fitted BFI value in a table that can be compared with the ideal ones. The details about the typical behavior of the measurement and the range of values can be found in Appendix C.

Similarly, for each probe position, a hundred consecutive measurements were averaged for TRS DTOFs. Afterward, the fitting procedure was carried out, and the physiological and optical parameters were obtained.^33^^,^^42^ To represent the raw data as normalized, the maximum of the DTOF is used for normalization (by simple division).^43^^,^^44^ Apart from a visual inspection, TRS curves were described in terms of their overall shape (qualitative), amount of broadening with respect to the instrument response function (IRF), and the dynamic range.^33^^,^^45^^,^^46^ Furthermore, we have quantitatively characterized the data by some descriptive parameters, such as relative full-width at half-maximum (rFWHM) of DTOF with respect to the IRF, difference of FWHM between DTOF and IRF (ΔFWHM), SNR, $\mu_{a}$, $\mu_{s}^{\prime }$, and ${\mathrm{StO}}_{2}$. The values for each case example are reported in a table divided per light wavelength, either 687 or 830 nm. The rFWHM was calculated as the ratio between the FWHM of the DTOF and the FWHM of the IRF, although more complex equations do exist for a more precise estimation, taking into account for example the laser power.^47^ The ΔFWHM, instead, was calculated as the difference between the FWHM of the DTOF and the FWHM of the IRF. The SNR was computed as the ratio between the maximum of the TRS DTOF curve divided by the standard deviation over a portion of the background signal (the noise) present before the light pulse itself. Again, details about the typical curve behavior and values range can be found in Appendix C.

As a side note, general backscattered light shapes and indicative optical values are valid when the diffusion approximation is valid, as well as the approximation to have greater reduced scattering coefficient over the absorption one (weakly absorbing and highly scattering media).^40^^,^^48^ On the contrary, other methods should be used to describe the diffused curve shape.^48^ Similarly, other approximations used to solve the diffusion models for DCS and TRS may not hold true (i.e., homogeneous model, slow temporal changes in the fluence rate, and random walk approximation).^42^ For this reason, both qualitative and quantitative analyses are helpful in the quality assessment of the results.

## 3 Results

### 3.1 Population Characteristics

Overall, data from 121 different probe positions from 36 subjects were included from June 2016 to October 2020. The distribution of the subjects among the cohorts is reported in Table 1 as well as the pertinent demographic and clinical information.

**Table 1:** Characteristics of the recruited patients are reported in this table. The parameters are expressed in proportions, such as males:females (M:F), yes:no (Y:N), and percentage over the total. Age is given as the mean [standard deviation (SD)] for the group. Subjects were recruited from three pathological categories or cohorts. TBI, traumatic brain injury; MCA, malignant infarction; SAH, subarachnoid hemorrhage.

Gender	M:F	27:9		
Age (years)	Mean (SD)	41 (15)		
Subjects per cohort	MCA	9		
	TBI	26		
	SAH	1		
Brain/skull surgery presence	Y:N	23:98	%	19
Skull presence	Y:N	108:13	%	89

The frequency of the different tissue composition categories (see above for the classification) is listed in Table 2. For three positions, the CT scan was not available to perform a precise assessment, but we could still obtain a proper signal and record the presence/absence of skull. Precisely, one of them was on a decompressive craniectomy area without underlying bone, whereas two were supposed to probe a healthy-like tissue, with skull and cortex below.

**Table 2:** Classification of all tissue compositions for the ROIs after the examination of the CT scans, when available, or the clinical information. Each tissue type is represented by initials and is described by going from the outer layer toward the gray matter as a sum of layers/components. Details about all acronyms are in the text. Two main categories are identified: having a regular ST as in the left column or an SST as in the right column. The tissue compositions denoted by an asterisk (*) do not include CB as a listed tissue type. The CT scans were not available in three ROIs to perform a precise assessment, which were omitted.

Regular subcutaneous tissue		Swollen subcutaneous tissue	
ST+CB+NB	14	SST+CB+NB	13
ST+CB+CSF/NB	7	SST+CB+CSF/NB	2
ST+CB+A/CSF+NB	3	SST+CB+CSF/A+NB	1
ST+CB+CSF+NB	22	SST+CB+CSF+NB	10
ST+CB+A+NB	2	SST+CB+A+NB	5
ST+CB+A+BC	1	SST+CB/A+NB	1
ST+CB+BC	2	SST+CB+BC	2
ST+CB+SAH+BC	2	SST+CB+CSF/SAH+NB	3
ST+CB+SAH+NB	6	SST+CB+EH+CSF	1
ST+CB+ICH/NB	1	SST+CB+EH+CSF+NB	1
ST+CB+IT	4	SST+CB	2
ST+A+NB*	1	SST+CB+EH	1
ST+EH+BC*	1	SST+CSF+CB/A	1
ST+EH+IT*	3	SST+EH+IT*	1
ST+EH+CSF/IT*	2	SST+CSF+NB*	1
ST+IT/NB*	1	SST+IT*	1

### 3.2 Case Examples

A table summarizing the following pathologic examples is provided in Appendix E. We suggest the readers to make this table available at the bedside to facilitate their reading and the comparison of cases.

#### 3.2.1 Healthy case H0

**Fig. 5:**
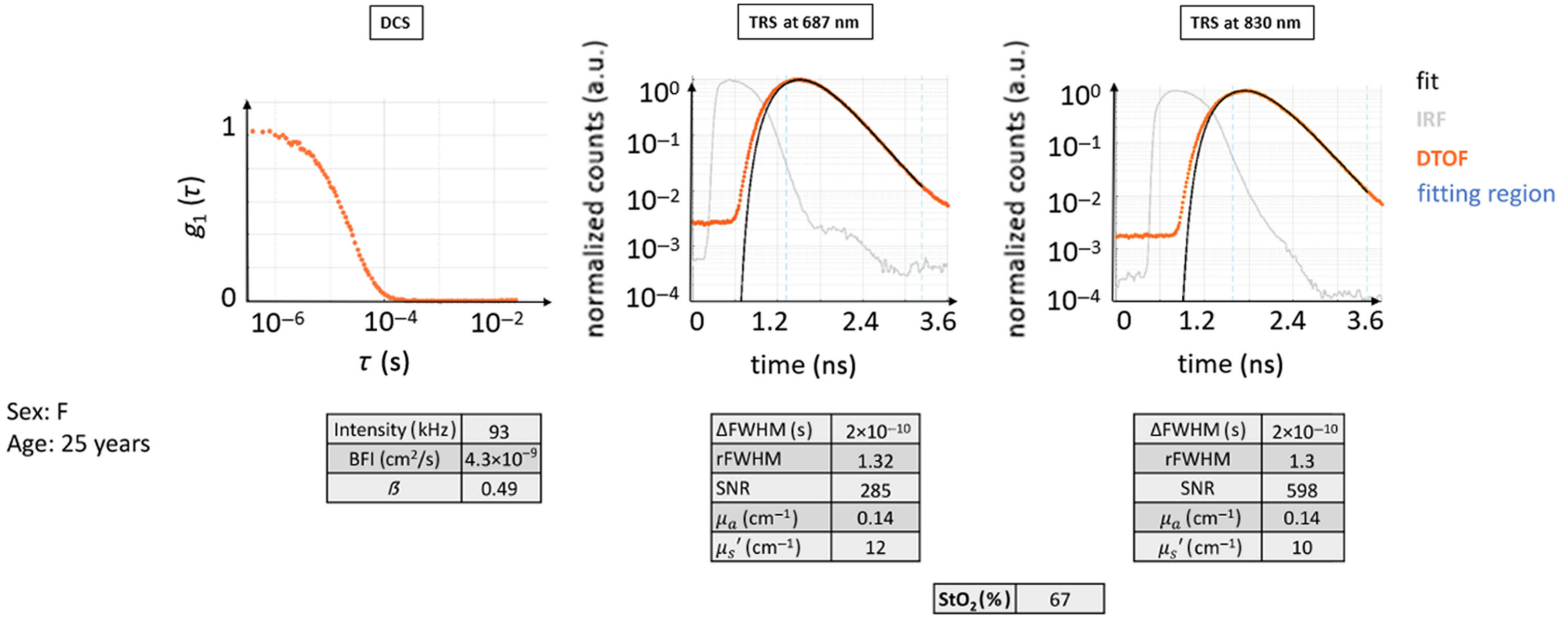
Typical curves for TRS and DCS measured on a healthy volunteer (healthy case H0). The DTOF curves and their fit are shown. The fitting region of the DTOF curves is indicated in light blue dashed lines. It is important to point out that in the DTOF graphs, the 0 ns on the time axis was arbitrarily set and does not coincide with the peak of the IRF curve to be able to show the entire curve and its initial background. The typical distance between the IRF curves and their diffuse DTOFs can be appreciated, as well as the slower linear decay of the tail of the DTOF for both wavelengths. The latter also presents almost a decade of higher background before the curve, with a consequently decreased dynamic range for the DTOF. The fit follows very closely the curves of raw data points. The autocorrelation curve also presents a typical behavior as described in the text.

This case illustrates the data from a healthy 25-year-old female subject (see Fig. 5) where both TRS and DCS curves possess the typical shape following their respective theoretical models.

In this case, the decay for DCS $g_{1}(\tau )$ is roughly exponential. Due to the normalization, the curve starts near one and approaches zero at $∼10^{-4}$ s. The intensity rate is high, above 90 kHz, with a β=0.49 and very close to ideal. The BFI value is within the normal range.

The TRS curves from both wavelengths show the expected behavior: the peak of the DTOF is shifted to later times with respect to the IRF peak, and the curve is broader with the FWHM of the DTOF, much larger than that of the IRF. DTOF plots are attenuated in amplitude than the IRF, with a long linearly decaying tail. The maximum signal level is acceptable, almost three decades above the noise background. The two wavelengths behaved similarly, and their shape looks alike.

The obtained $\mu_{a}=0.14\;{\mathrm{cm}}^{-1}$, $\mu_{s}^{\prime }=10-12\;{\mathrm{cm}}^{-1}$, and ${\mathrm{StO}}_{2}$ around 67% are not far from the indicated ideal values.

Additional similar examples are provided in Appendix D.

#### 3.2.2 Case 1

**Fig. 6:**
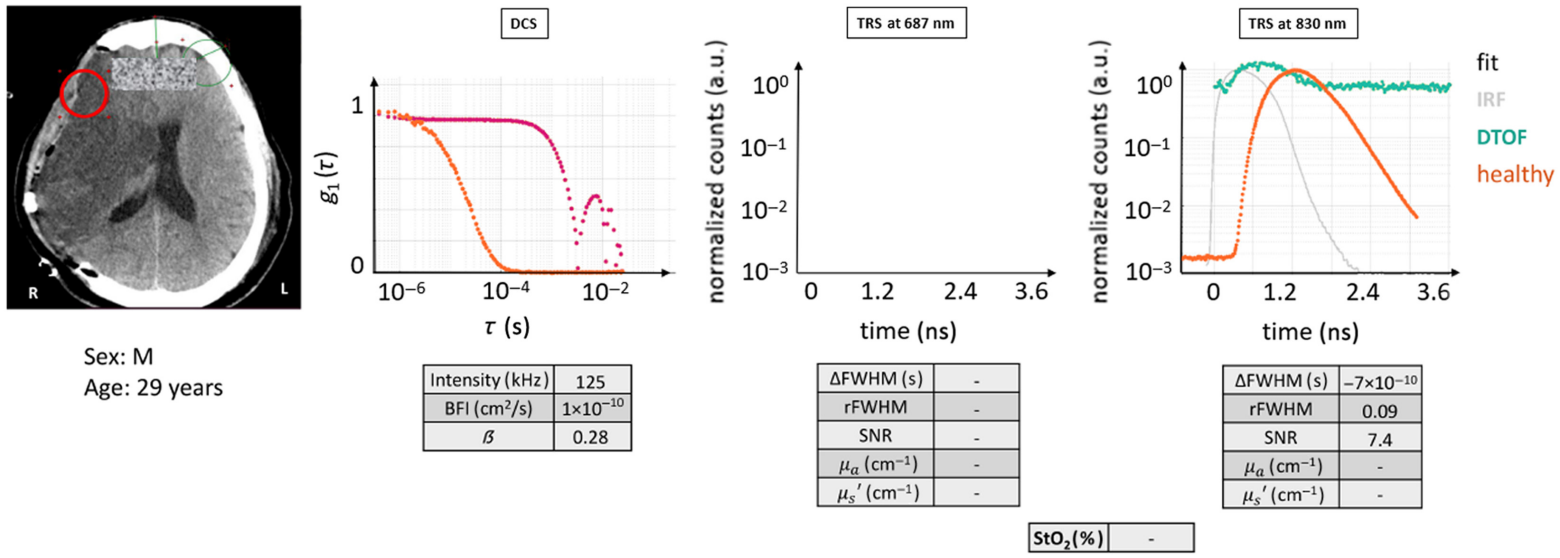
Case example 1: acquisition on an infarcted area after hemi-craniectomy. DTOF at 830 nm and autocorrelation curves for the red circled placement on the CT scan are displayed. The latter is marked by a slow decay and the former by very poor SNR. The DTOF at 687 nm is missing due to even poorer data quality. The figures of merit are tabulated. Red circle: ROI (Ø30  mm). Magenta curve: current subject’s $g_{1}(\tau )$ curve.

In this patient (male, 29 years), the probe was placed above a region that underwent decompressive craniectomy after malignant MCA infarction. The tissue composition was ST + EH + IT. The qualitative evaluation is based on Fig. 6. As seen in the figure, the results from 687 nm were not reported because there were very few detected photons. This is most likely due to the presence of highly deoxygenated blood in the infarct zone which absorbs differentially higher in this wavelength. This is coupled with the fact that the missing skull implied that the probe was resting on the brain/muscle directly, which absorbs more light than the typical scenario. Given the performance of this study’s device, the number of photons detected by the TRS for 830 nm was very minimal presumably due to the same reasons as above. The minimal DTOF curve that was observed could not be reliably analyzed with a photon diffusion model because it did not indicate a scenario where scattering dominated over absorption. Therefore, no optical properties are reported.

DCS results also showed an atypical behavior that is often observed on dead tissue or on regions with minimal red blood cell movement.^3^ Unexpectedly, the intensity level was quite high, but the β parameter was low and the decay time (τ) was quite long lasting longer than $10^{-3}\;s$, implying very low BFI. This may be expected because infarcted tissue is expected to have lower blood flow. On the other hand, the model did not fit the curve well and the quantitative results cannot be trusted. Note that once the fitted BFI is lower than $3\times 10^{-10}\;\mathrm{cm}/s^{2}$, the fits may be unreliable because the ergodicity of the measurement may no longer be sufficient due to very low blood flow.

In a case scenario like this, few considerations can be made to yield some guidelines. As for TRS, because the SNR is low, summing up more curves than a hundred could be a method to improve the DTOF or a longer acquisition time. Of course, higher injected source power and/or a larger detection area would also help. However, even with these improvements, it may not be sufficient to obtain a good fit by the diffusion model unless a device with a much narrower IRF is utilized, and the data are fitted by a more complex model (higher order approximations to the radiation transport equation or a Monte Carlo method^30^^,^^31^). For DCS, the current practice is to report this type of curve as being indicative of blood flow less than one percent of the normal without a specific value.

As a consequence, quantitative results cannot be obtained by diffuse optical methods, but the nature of the data, especially the DCS data, reveals minimal blood flow in the region. Unfortunately, it is hard to predict how this type of tissue would reveal itself to a multi-distance CW NIRS device.

#### 3.2.3 Case 2

The ROI delineated in this case shows an area of the brain where extracerebral muscle tissue was swollen following a craniotomy, where the bone was repositioned in the head after surgery to treat a lesion (tissue composition: SST + CSF + CB/A). The patient (male, 18 years, initial GCS = 5) presented a diffuse axonal injury with epidural hematoma in the left cerebral hemisphere and cranial fractures due to a car accident and had undergone a decompressive craniectomy on the injured hemisphere.

**Fig. 7:**
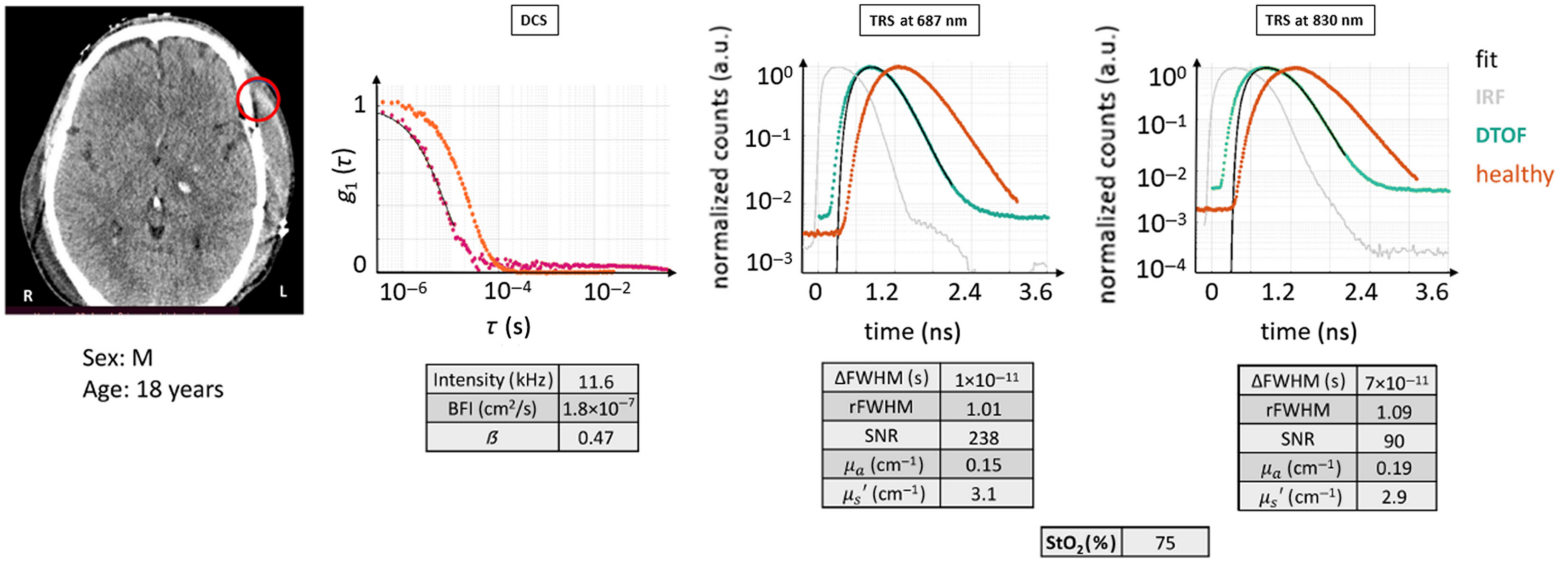
CT scan with the ROI marked in red for case example 2, with the acquisition on a swollen muscle. DTOF curves and autocorrelation curve are compared with the respective ones from case H0, in orange. Lower intensity for all methods and greater/faster diffusion can be seen. The figures of merit are tabulated. Red circle: ROI (Ø30  mm). Magenta curve: current subject’s $g_{1}(\tau )$ curve.

The raw data and the clinical conditions are showcased in Fig. 7. For the depicted ROI, there is a high probability that the brain was not actually reached by the diffuse optical light path.

DTOF curves qualitatively appear similar for the two wavelengths: their dynamic range looks similar to the one expected from a healthy tissue, and the DTOF appears broader than the IRF and with a slower linear decay. The DCS curve, instead, had a faster decay toward 0 intensity than the given healthy example, implying a faster BFI, although the curve shape is similar to a healthy-like one and its fitting looks reliable. In quantitative terms, the DCS detected a low light intensity rate, whereas the autocorrelation function had a comparable β coefficient and a higher BFI with respect to the healthy example. $\mu_{a}$ coefficient was high for both DTOF curves, which is expected due to the faster linear decay, whereas $\mu_{s}^{\prime }$ was quite low. On one hand, the latter may be due to an amount of direct light, which could appear during hand-held measurements, where the adherence of the probe to the skin depends on the person who holds the probe. It could be remedied with the use of common load sensors, for example. On the other hand, swollen muscle tissue is characterized by accumulated liquid, which is mostly transparent/translucent and scatters less.

If these results are evaluated without care, they could be interpreted as representing true cerebral hemodynamics. However, from the CT scan, it appears evident that the probed area had a low probability to have intersected the brain cortex: the atypical increased thickness of the upper layers implies that the bulk of the photons did not reach the brain. In light of this, these data have to be interpreted as invalid for measuring the brain. Rather, they reflect the properties of the swollen muscle and, partially, the bone, which could be relevant for other reasons or studies.

From the perspective of the TRS, it is possible that a better setup, with a higher laser power and tighter FWHM of the IRF could allow us to time gate the photons and retrieve the information from the deeper layers. This would also require a more complex analysis method, e.g., using layers or anatomical priors, which are known to be unstable.^29^^–^^31^

#### 3.2.4 Case 3

**Fig. 8:**
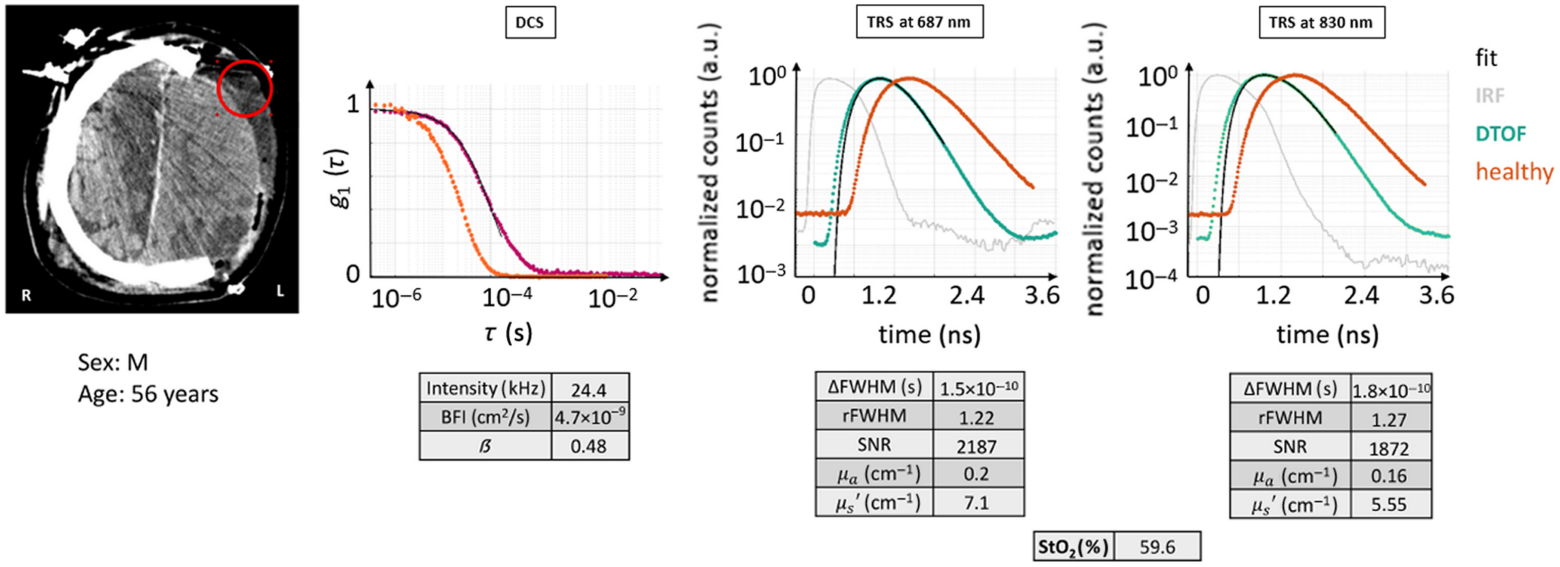
CT scan with the ROI marked in red, DTOF curves, and autocorrelation curve for case example 3: acquisition on a swollen muscle after a craniectomy. All figures of merit are tabulated. The decay for the autocorrelation curve is slower than in a healthy condition, whereas the DTOFs’ diffusion is lower, due to the lack of skull-related scattering events. Red circle: ROI (Ø30  mm). Magenta curve: current subject’s $g_{1}(\tau )$ curve.

Figure 8 shows the findings from case 3, which is a common, yet very difficult case to interpret. The data were acquired above a swollen muscle with large edema and after a craniectomy procedure (tissue composition = SST + CSF + NB, male patient, 56 years). The etiology was a fall (initial GCS =13, later reduced to 8 at intubation) that produced subdural hematoma and subarachnoid hemorrhage requiring a decompressive craniectomy procedure on the left cerebral hemisphere.

At the TRS level, the DTOF curves detected for both wavelengths are analogous: sufficient dynamic range in amplitude, shorter photon path lengths compared with a typical brain, and fast linear decay of the backscattered light pulse tail in line with a higher absorption value which is due to the lack of the skull. Numerically, the values found display an absorption coefficient higher than usual, comparable to the previous case 2, whereas the $\mu_{s}^{\prime }$ is intermediate between case 2 and a normal expected value. DCS shows a slower decay and comparable shape to the normal case. Quantitatively, the DCS intensity of the acquired curves was acceptable, ∼24  kHz averaged over four channels, whereas β was comparable to a healthy-like case. Finally, the obtained BFI is lower than for an intact head condition.

Referring to the CT, the photons have a low probability to have crossed their path with the cerebral gray matter, due to the thickness of the outer inflated tissue. Therefore, similar conclusions and suggestions to case 2 could be made here with a main difference: the absence of skull, which may account for the differences in the scattering properties and lower BFI.

This is another case of data appearing “normal” while possibly not reflecting the cerebral hemodynamics. The CT scans should be considered when evaluating these results.

#### 3.2.5 Case 4

**Fig. 9:**
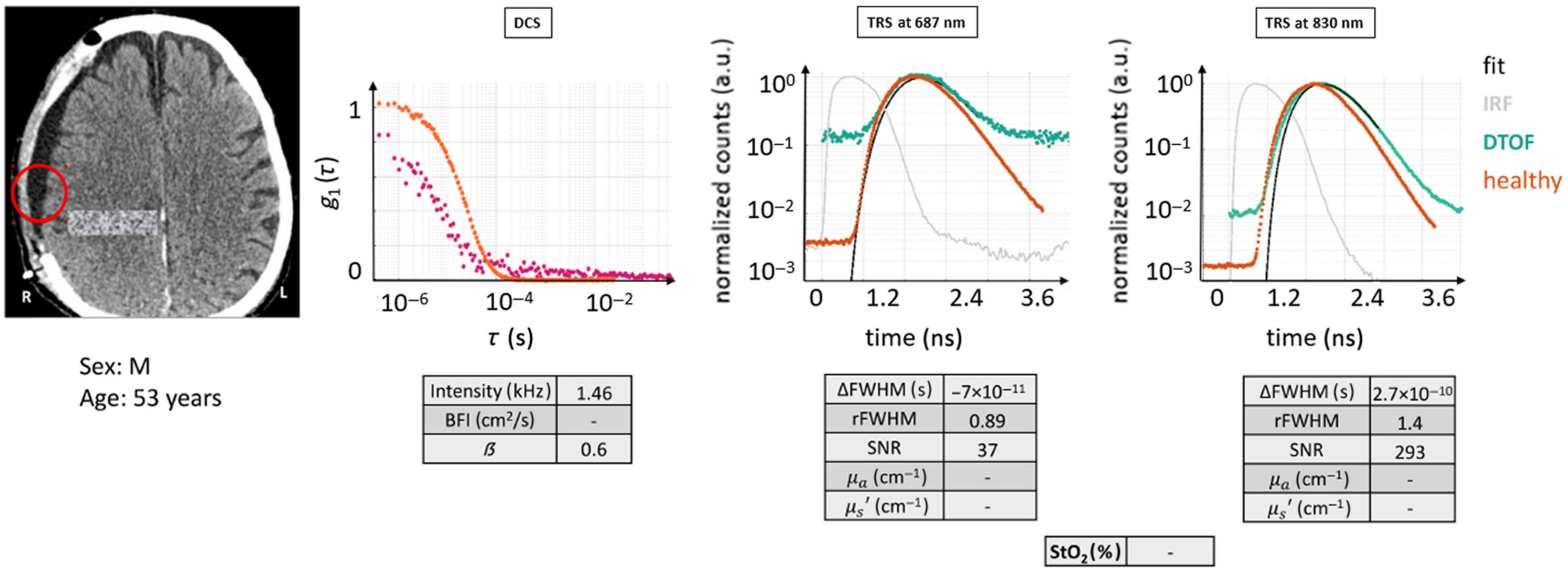
CT scan with the ROI marked in red, DTOF curves, and autocorrelation curve for case example 4: acquisition on a CSF accumulation after a craniectomy. The presence of inflamed tissue, CSF, and cerebral cortex has the combined effect of reducing at minimum the intensity rate of the autocorrelation function, not allowing for a reliable fit, and of the 687 nm DTOF, with just one decade of dynamic range. It is difficult to interpret the 830-nm DTOF curve as extraneous. The available figures of merit are tabulated. Red circle: ROI (Ø30  mm). Magenta curve: current subject’s $g_{1}(\tau )$ curve.

In this case example, the probe was lying upon a region that underwent a decompressive craniectomy with an accumulation of cerebrospinal fluid between a thin layer of muscle and the brain, as visible in Fig. 9. The patient (male, 53 years) had an ischemic lesion on the right capsule–thalamic region and a hemorrhagic frontoparietal contamination on the left cerebral hemisphere after a fall (GCS = 11). He was treated with a decompressive craniectomy on the right hemisphere. Despite what is shown in the figure, the skull fragment had been replaced to its original position at the time of the measurement, and therefore, the tissue composition beneath the probe was SST + CB + CSF + NB.

Qualitatively, DTOF curves reveal that overall, there were fewer detected photons but with comparable light diffusion to the healthy tissue. The 687-nm backscattered light pulse is more affected than the 830 nm one, which presumably is due to highly deoxygenated hemoglobin presence (which absorbs more at 687 nm). The data could not be fitted to the model with the current set-up and acquisition conditions.

DCS was unable to detect a usable intensity. Even after a long integration time, the curves shown in Fig. 9 are quite suspect, likely reflecting structures of noise (e.g., after-pulsing).

CSF is mostly translucent and low scattering could lead to a lower number of photons being scattered back to the detector. Furthermore, this measurement was taken over a region where the hair was shaved but hair follicles remained in place. This complicates the data acquisition greatly. In this case, the effects are quite visible and even without the CT scans one could notice the poor quality of the measurements. This may or may not be true for multi-distance CW measurements—stressing one more time the importance of the consideration of the underlying anatomy.

#### 3.2.6 Case 5

**Fig. 10:**
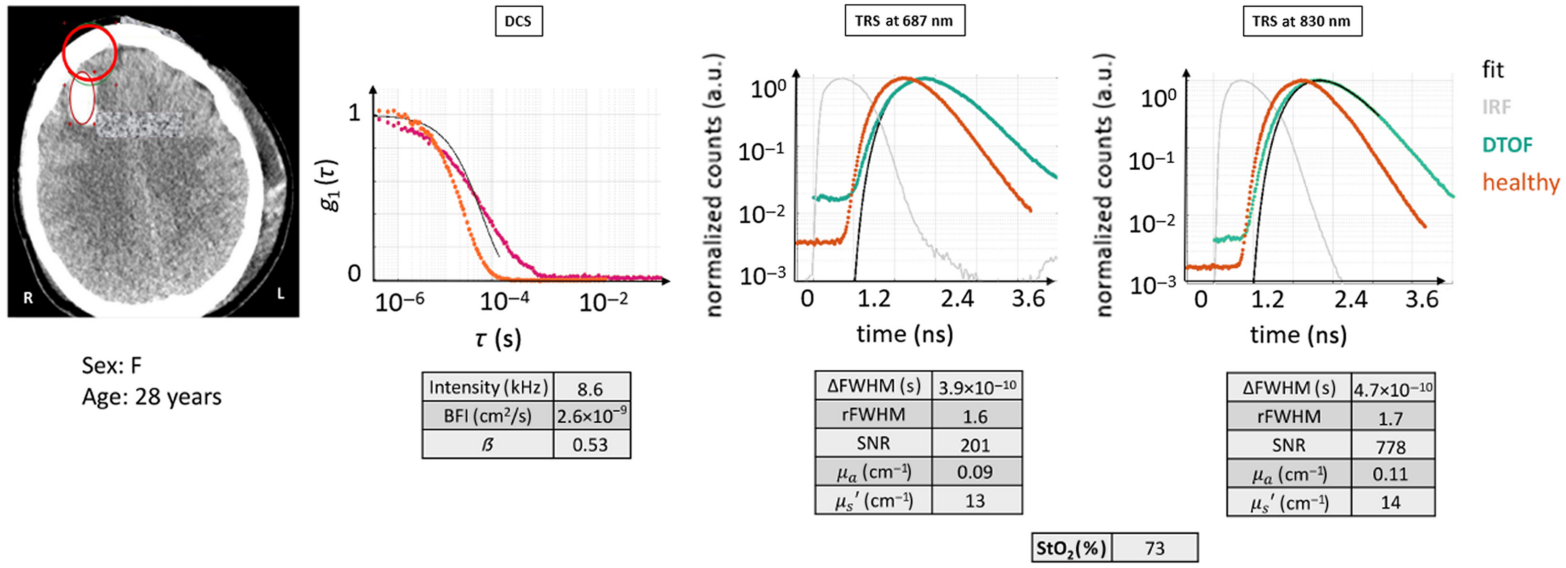
CT scan with the ROI marked in red, DTOF curves of TRS, and DCS autocorrelation curve for case example 5: acquisition partially intersecting a hemorrhage. Both DTOF curves and values resemble the healthy example of case H0, making it hard to distinguish them apart from the decay, which appears slower for all methods. The figures of merit are tabulated. Red circle: ROI (Ø30  mm). Magenta curve: current subject’s $g_{1}(\tau )$ curve.

As highlighted in Fig. 10, this case offers an example that features a partial intersection of an intracerebral hemorrhage, which is the large, oval bright area in the CT scan enclosed in the red oval. Most likely, the measurements mainly reflect the skull and gray matter but could be influenced by the hemorrhage and its surroundings to some degree. The tissue composition assigned to this probe location is ST + CB + ICH/NB. The patient (female, 28 years) suffered a TBI and SAH (diffuse lesion type III) along with a complete spinal lesion at the C3 level. A decompressive craniectomy was performed on her left cerebral hemisphere.

By inspecting the DTOF curves, at a qualitative level, both wavelengths possess the same features: wide dynamic range and dispersion of the curve that is comparable to a normal tissue-like, slower linear decay which should mean lower absorption properties. DCS autocorrelation function, instead, has a damped shape, flat in the initial part, which does not appear to reflect a single exponential and has a slower decay.

On the quantitative side, the TRS figures of merit feature: $\mu_{a}$ around 0.1 to $0.09\;{\mathrm{cm}}^{-1}$ which are typical for a healthy head, high $\mu_{s}^{\prime }$, high SNR, and good fit to the model. However, the ${\mathrm{StO}}_{2}$ appears to be ∼10% higher than that for a healthy brain. Conversely, the DCS curve is characterized by low intensity and high β, which was probably affected by the typical afterpulsing observed in this class of photon counting detectors, i.e., in a simplistic manner, afterpulsing is the detection of a second, false photon by the detector after the initial photon in a probabilistic fashion (see details in C). This led to the observation that the fitting procedure ended up with a BFI value much lower than usual, but because the data are dominated by noise, this is not a reliable value.

Under these circumstances, a blind measurement analysis could lead to the wrong conclusion: the hemorrhage is there, but the way in which it influenced the signal is unclear. In conditions of reasonably maintained blood flow, low partial pressure of tissue oxygenation (${\mathrm{PtiO}}_{2}$) readings are reported in the literature,^49^ which are a symptom of low oxygen extraction by the tissues. Therefore, higher than normal (i.e., greater than 70%) tissue oxygen saturation is expected in the blood, which is confirmed by this measurement.

The $g_{1}(\tau )$ DCS curve is certainly affected by the hemorrhage indicating a heterogeneous result—possibly a probed volume with large volumes of low and high scatterer dynamics leading to a complex shape.

Overall, if the presence of the hemorrhage is taken into account, it may be possible to select a different region to either try to obtain brain values or to get information reflecting the hemorrhagic region itself.

#### 3.2.7 Case 6

**Fig. 11:**
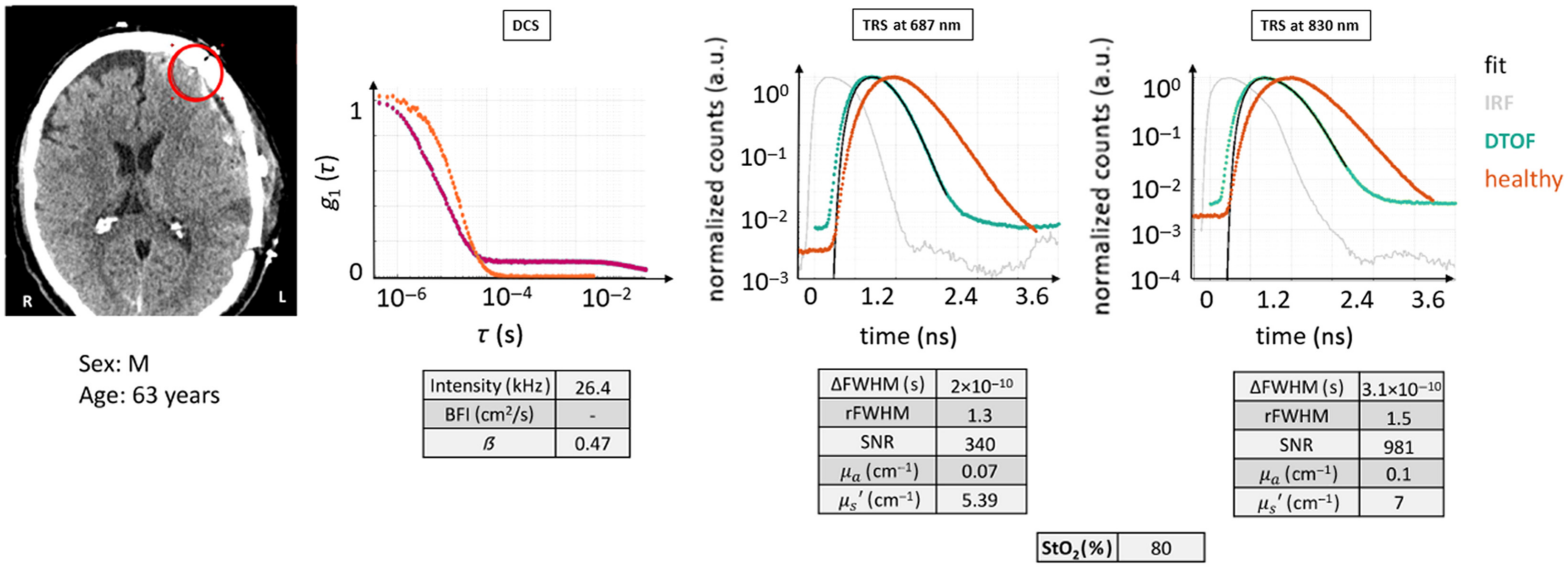
CT scan with the ROI marked in red, DTOF curves, and autocorrelation curve for case example 6: acquisition partially intersecting a hemorrhage. This case should, in principle, be relatable to case example 5. However, the intersected area is more diverse, and the effect on the optical signals appears quite different: a faster decay for the DTOFs and an autocorrelation curve that does not reach 0. All figures of merit are tabulated. Red circle: ROI (Ø30  mm). Magenta curve: current subject’s $g_{1}(\tau )$ curve.

The case example presented in Fig. 11 is similar to the previous case: the probe was positioned over the skull in a region that included tissue with a subdural hemorrhage. In fact, this patient (male, 63 years) had experienced a fall from 2 m of height that led to a contusion (GCS = 14) and following lesion on the left cerebral hemisphere necessitating a craniotomy procedure to extract the hematoma on the temporal left side of the head. The probed region was assigned ST + CB + SAH + NB as tissue composition.

The DTOF curves show a faster linear decay than a healthy tissue, with sufficient dynamic range and comparable dispersion with respect to their IRF for both of the wavelengths. On the DCS side, the initial part of the intensity autocorrelation curve is similar to a healthy-like case, but its final portion did not properly reach 0 after the exponential decay, rather it presents a secondary exponential decay. This is most likely indicative of a shallow region with low blood flow.

Quantitatively, on the TRS side, the SNR is high, and the relative and difference of FWHM agree with the expectations for a diffused DTOF for both wavelengths. Moreover, the data agree very well with the model. Interestingly, the information found is relatable to the previous example. In this ROI, the $\mu_{a}$ values are still low, between 0.07 and $0.1\;{\mathrm{cm}}^{-1}$, ${\mathrm{StO}}_{2}$ is quite high (80%), whereas $\mu_{s}^{\prime }$ coefficients are $∼6\;{\mathrm{cm}}^{-1}$, lower than healthy tissue-related ones.

The DCS curve deviated from the homogeneous model significantly, although the intensity rate was decent and β in line with normal values. Therefore, the BFI cannot be considered reliable. It is also worth noting that the final part of the curve affects the calculated numbers due to the physics, even if the cutoff threshold for the fitting procedure of the DCS curve occurs at an autocorrelation value of 0.3. Moreover, the second exponential decay in the tail of the autocorrelation function may suggest that the method is detecting atypical tissue, as if a sudden change in the morphology creates a second layer that abruptly modifies the decay.

This case is difficult the evaluate. Clinical interpretation of the CT scan reveals significant heterogeneity and abnormalities under the probe, and the optical data show deviations from the physical models for the DCS curves. A conservative approach would err toward invalidating the optical measurements, whereas it surely highlights potential issues with CW measurements, which may not reveal these problems so clearly.

#### 3.2.8 Case 7

**Fig. 12:**
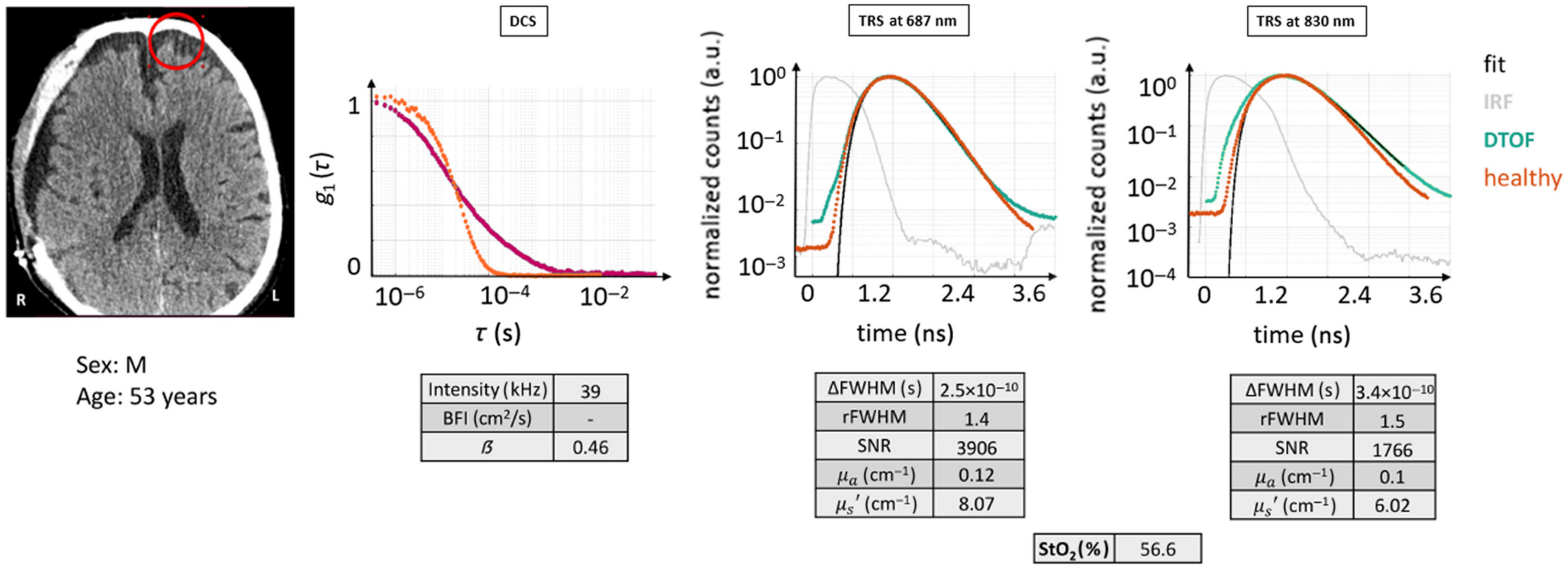
CT scan with marked the ROI in red, DTOF curves, and autocorrelation curves for case example 7: acquisition on a CSF accumulation below the skull. For the DTOF curve, the similarity to the healthy case H0 makes them almost impossible to tell apart. On the contrary, for the DCS curve, the slower decorrelation toward zero and the shape seems to be affected by the CSF composition. The figures of merit are tabulated. Red circle: ROI (Ø30  mm). Magenta curve: current subject’s $g_{1}(\tau )$ curve.

Another possible situation is represented by the presence of accumulation of CSF between the skull bone and the brain, as shown in Fig. 12. The patient for this case was the same one as in case 4. The tissue composition for this ROI is ST + CB + CSF + NB.

Observing the DTOF curves, it is immediately evident that they have an appearance that resembles that of normal tissue: the dispersion, the dynamic range, and the shape are very similar. On the DCS side, the shape is highly distorted, possibly revealing multiple decay constants.

Also, at a quantitative level, the values are in line with an acquisition obtained from a healthy head for the TRS, although resulting in a low ${\mathrm{StO}}_{2}$. For the DCS, the models cannot be used to fit the data.

This case is particularly problematic when interpreting the data and the worst is that, from a careful evaluation of the quality of the TRS data, it is practically impossible to notice that something is hidden behind the findings. As previously mentioned, CSF is a fluid with low scattering and absorption properties. It appears that the presence of CSF is tricky and difficult to recognize, especially considering that it is only an intermediate layer in this measurement. Moreover, considering that, given the thickness of the intermediate layers, the gray matter may have not been reached, it is reasonable to obtain a low tissue saturation. In this context, the CT scan is a primary source of information. A pre-measurement view of the CT scan should not be omitted in this case.

#### 3.2.9 Case 8

**Fig. 13:**
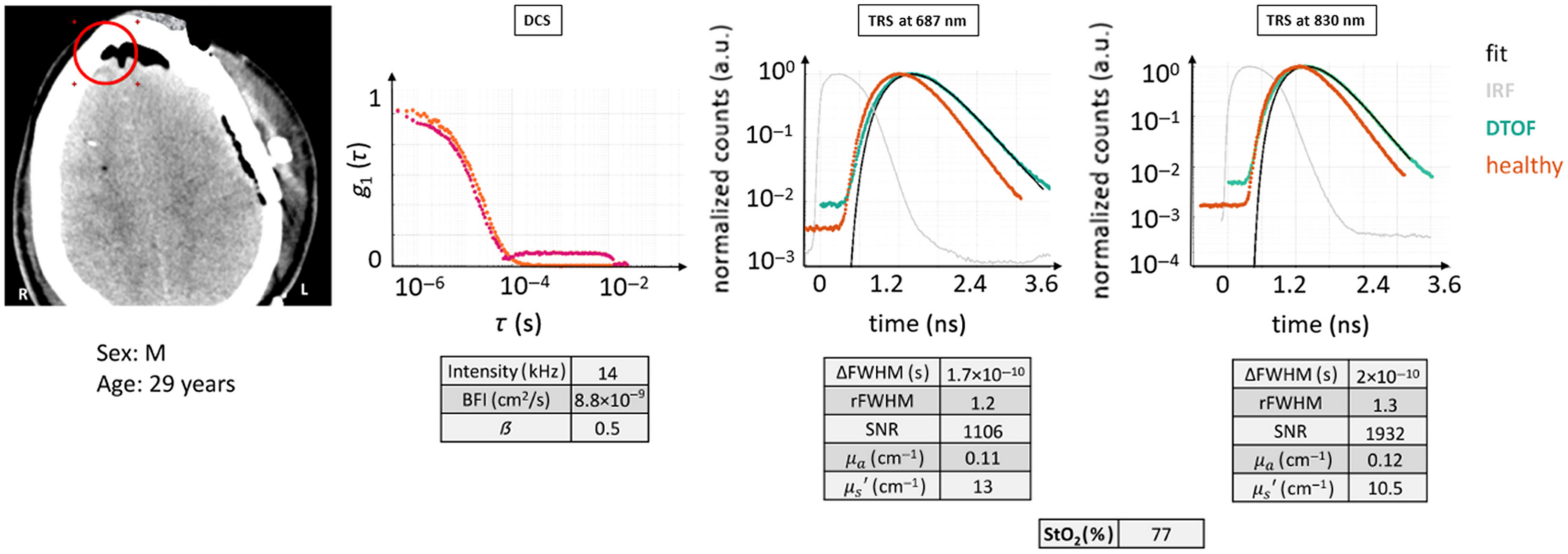
CT scan with the ROI marked in red. DTOF curves and autocorrelation curve for case example 8, where the probe was above a region filled with air trapped below the skull. As for the initial portion of the autocorrelation curve, the resemblance with the healthy tissue one is high, whereas this is not true for the final decay, which is actually more related to scattering events. The figures of merit are tabulated. Red circle: ROI (Ø30  mm). Magenta curve: current subject’s $g_{1}(\tau )$ curve.

Fig. 13 shows an example in which, after an injury, air got trapped between the skull and the frontal lobe of the brain. This is a quite common situation, especially after a concussion and after a surgery,^50^ known as pneumocephalus. Moreover, there is a natural cavity, the sinuses, which are at times more pronounced in some people and that can be present up to a couple of centimeters above the eyebrow line. In this case example, the patient (male, 29 years) had TBI (initial GCS = 3) caused by a fall that led to a therapeutical decompressive craniectomy on the left cerebral hemisphere. The indicated ROI also appears to include the brain and skull, with a tissue description as SST + CB + A + NB.

The qualitative analysis of the TRS curves reveals a very strong analogy with a healthy head situation, even if the linear decay is a little less rapid. For the DCS curve, on the other hand, the situation is similar to that of cases 6 and 8, where a second decay appears in the final part of the curve.

In quantitative terms, $\mu_{a}$ coefficients appear normal, whereas ${\mathrm{StO}}_{2}$ and $\mu_{s}^{\prime }$ higher than for a healthy head. As regards the $g_{1}(\tau )$ curve, the intensity rate is quite reduced (14 kHz), but β and BFI values seem reasonable, with a proper fit, and comparable to a healthy scenario.

Consequently, it looks like diffuse optics cannot clearly distinguish between a normal head and one where air is comprised. Most likely, the presence of a region with trapped air, a non-diffusive medium, led to a complex result that could have confused traditional quality control methods.

### 3.3 Dataset Description

Pertaining to the patient data spreadsheet, 121 rows for a different cerebral position (ROI) were included, with a total of 242 rows, because the optical properties were reported separately for the two wavelengths of the TRS sources. Please note that some numbers, although unusual for the normal cerebral tissue, were still kept because the criteria for rejection did not apply nor did the data fitting procedure fail.

Concerning the MATLAB-structured data files, they were gathered for 91/121 ROIs. In fact, we have omitted the data for the 30 measurements that were obtained with a different device, as explained in the methods, due to the difference and complexity of the files. However, these data were used for the averages and case examples and are present in the other formats.

As for the CT scan images, no CT was identified as close enough in time to be used for the proper tissue identification or was not provided for three measurement sessions with a total of six ROIs.

The created database is available on CORA, Repositori de dades de Recerca repository (Repository Technology/Platform: Dataverse) at https://doi.org/10.34810/data1355, where the CT scans are available upon request to the authors.

We believe this database can be valuable to other researchers. For instance, for replication or validation, by reprocessing the data with their own data processing methods, or analyzing them from a different perspective. Moreover, data integration between the anatomical data provided by the CT scans and the optical data could be performed to more accurately unveil the optical properties of the atypical tissue structures and layers as well as to simulate the light propagation through a head with a heterogeneous structure, taking advantage of these anatomical priors.

### 3.4 General Recommendations for Optical Measurements with Examples from Literature

We now speculate about practical recommendations for quality control in hybrid diffuse optical neuro-monitoring as were already partially outlined in the case examples above. It is important to note that these guidelines are not only based on the few case examples shown in this paper but also on the authors’ experience and the literature.

It could be said that there are three main categories of guidelines for improving measurements on MCA, SAH, and TBI patients:

1. the employment of more sophisticated and powerful diffuse optical devices
2. the use of more correct or more complex methods of analysis, applying corrections to the standard model generally used
3. more accurate positioning of probes to avoid regions that can contaminate the information sought.

We have gathered this information in Tables 3 and 4, without the intention of compiling a complete review.

**Table 3:** Set of recommendations and guidelines: part 1. (1) Device performance limitations, (2) loss of data due to model failure due to morphology/poor quality, and (3) poor/inaccurate probe positioning.

Problem category	Problem	Technique	Solution/recommendation	References
(1)	Insufficient signal intensity by device component and not due to morphological structures	TRS	Higher SNR by greater laser source power (i.e., greater than 5 mW for TRS) and lower background noise of the detector for an improved dynamic range	Pirovano et al.,^51^ Buttafava et al.,^52^ and Ban et al.^53^
(1)	Reduced signal intensity by device component and not due to morphological structures	DCS	Higher SNR by greater laser source power conveyed by double sources or wider source areas and increased detection channels	Cortese et al.,^54^ Carp et al.,^55^ and Liu et al.^56^
(1)	Morphological structures “limiting” the signal due to their unusual optical properties	TRS	Implement threshold values (i.e., for the number of photons, total hemoglobin concentration changes against the contrast, instability of the laser peak over time, water content, and coefficient of variation of different parameters) to guarantee sufficient data quality	Giovannella et al.^57^ and Giacalone et al.^58^
(1)	Morphological structures “limiting” the signal due to their unusual optical properties	TRS	Tighter FWHM of the IRF (∼100 ps)	Pirovano et al.,^51^ Buttafava et al.,^52^ and Ban et al.^53^
(1)	Lack of slow frequency content in the signal (i.e., slow waves and pulsatility waveform)	TRS and DCS	Fast data acquisition (>20 Hz)	Liu et al.,^56^ Ruesch et al.,^59^ and Parthasarathy et al.^60^
(1)	Limited dynamic range and distortion effect typical of single-photon detectors (pile-up effect)	TRS	Implementation of fast-gated techniques to improve the dynamic range of TRS devices	Tosi et al.^61^ and Dalla Mora et al.^62^
(1)	Poor quality, consistency and reproducibility across experiments and setups, and lack of standardization	DCS	Follow “recipes” for DCS setups and acquisition settings (optimize count rate, averaging time, and variability in the initial optical data)	Cortese et al.^63^ and Carp et al.^55^

**Table 4:** Set of recommendations and guidelines: part 2. (1) Device performance limitations, (2) loss of data due to model failure due to morphology/poor quality, and (3) poor/inaccurate probe positioning.

Problem category	Problem	Technique	Solution/recommendation	References
(1)	Low accuracy and precision and lack of standards leading to poor reproducibility of the results	TRS and DCS	Simulations of precision and accuracy to establish target features for diffuse optical devices	Ntziachristos and Chance,^64^ Alerstamet al.,^65^ and Giovannella et al.^57^
(1)	Lack of consistency and reduced comparability across groups/devices and unreliable and irreproducible results	TRS and DCS	Minimize error and variability in the results by standardized experimental design	Giovannella et al.^57^ and Giacalone et al.^58^
(2)	Inaccurate modeling of light transport in heterogeneous tissues leading to inconsistency between computational models and experimental data	TRS and DCS	Algorithm improvement: heterogeneous structure incorporation/anatomical structures by Monte Carlo	Choi et al.,^66^ Francis et al.,^67^ Gagnon et al.,^68^ and Selb et al.^69^
(2)	Inaccurate modeling of light transport in the brain neglecting the impact of CSF thickness	TRS	Algorithm improvement: Monte Carlo simulations with layers and different CSF thicknesses	Ogoshi et al.^24^ and Ancora et al.^28^
(2)	Mixed contribution to signals from different structures and layers affecting the specificity of protocol responses	TRS and DCS	Double or multiple layer analysis (i.e., separation of intra- and extra-cerebral layers)	Gagnon et al.,^7^ Farina et al.,^32^ Ancora et al.,^28^ Young et al.,^23^ Zhao et al.^27^ Baker et al.,^70^ Li et al.,^71^ Wu et al.,^72^ and Verdecchia et al.^26^
(3)	Inconsistent probe placement across studies, limited reproducibility of DOS/TRS studies, and potential for misinterpretation of DOS/TRS findings	TRS and DCS	Probe placement guide and after measurement position record by CT scans, magnetic resonance imaging, positron emission tomography, palpation/visual inspection of the skin/head, and neuronavigators	Rao et al.,^73^ Giacometti and Diamond,^74^ Aasted et al.,^75^ Wu et al.,^76^ and Giacalone et al.^77^
(3)	Artifacts caused by external light or movement, reduced sensitivity due to sweat between probe and skin, and subjects’ discomfort	TRS and DCS	Probe fixation improvement	Büchner et al.^20^ and Zhao et al.^78^
(3)	Spurious signal arising from external light, lack of optimal probe-skin contact, and poor reliability and data quality	TRS and DCS	Real-time feedback information about the quality of attachment of the probes to the skin, i.e., by implementing sensors that detect anomalies and alert the user	Renna et al.,^79^ Giovannella et al.,^80^ Zanoletti et al.,^81^ and Rickard et al.^82^

In the present study, improvements in the DCS hardware were actually already achieved with respect to the currently available devices, as suggested by Carp et al.,^55^ where the power delivered on the head was high thanks to the double-source solution. Moreover, field programmable gate array–based correlators with faster and more reliable acquisition were used instead of the commercial ones, allowing for fast DCS acquisitions, and an increased number of detection channels, that enhanced the SNR.^54^^,^^83^

As a side note, some of the cited references have implemented solutions that could improve TRS and DCS issues in this context, but that are not currently implemented in these techniques.

However, despite these recommendations, sometimes it is unavoidable to obtain non-optimal measurement conditions, and it is, therefore, necessary to implement data quality checks, which are less important in cohorts of healthy subjects. Blindly fitting the TRS and DCS curves is risky and may lead to the loss of the hidden information. Quality checks can be implemented on simple figures of merits, such as the ones that were used in the case examples of this paper and the ones presented in Refs. 57, 58, 63, and 77. Such figures included the minimization of the least square error between data and fitted model, minimum total number of detected photons, difference in the temporal position between IRF and DTOF, and measurement reproducibility on a calibrated phantom.

## 4 Discussion

Several reasons are listed for the lack of widespread implication of NIRS-based monitoring in TBI. Among them, there are contamination from extracranial or extracerebral tissues, difficult implementation due to scalp and facial injuries, confounding effects caused by subdural, epidural and extracranial hematomas, and dead tissue.^18^^,^^18^^–^^20^^,^^84^^,^^85^ However, improvements in the computational modeling of DO and its integration with subject-specific imaging do represent a clear possibility to expand its use in brain pathology assessments.

In this work, we have characterized structural and optical signal abnormalities in severe brain injury, malignant MCA, and SAH patients. Overall, the results described problematic tissue types and how they affected the DO signals in a major attempt to understand if certain impaired brain regions are measurable and to promote data quality examination. In fact, the complex multi-layered structure of the adult human cranium already sets a limit to the accuracy of NIRS.^86^^,^^87^

We have recruited a pool of 36 patients that included 9 malignant MCA, 26 TBI, and 1 SAH. In 19% of the cases, surgery was performed on the subject, allowing for thirteen measurement acquisitions in the absence of skull. The surgical procedures included decompressive craniectomy, craniotomy, and resection.

Initially, we have described eight case examples with pathologies and one case of a healthy subject qualitatively and quantitatively. The selected cases had the objective of showing typical situations that one can meet when this type of patient is recruited and to provide a fairly representative sample, albeit limited to the variety of measurements that have been obtained. In some cases, the simple view of the curves made it possible to immediately understand the difference compared with the healthy case, whereas in others, it did not. Moreover, even at the level of numerical values, it was at times impossible to discern whether it was a curve generated by healthy or damaged tissue. Later on, some guidelines for measurement practice were outlined and related to the literature to provide insights and practical examples.

To the authors’ knowledge, there is no similar attempt in other studies on head-injured subjects and by hybrid DO technique. Some existing work regarding healthy subjects has been undertaken in, for instance, Refs. 88 and 77. In the latter, a strict quality control practice was also set,^77^ partially as we aimed to discuss in this paper. Some studies have reported oddities when CW-NIRS devices were used with hematomas, craniotomies, and craniectomies^20^^,^^23^^,^^89^^,^^90^ but did not inspect the cases. Other studies rather focused on the detection of hematomas, either by bi-hemispheric cerebral comparison or absorption values,^67^^,^^87^^,^^91^^–^^93^ by standard CW monitors. Instead, the effect of CSF has been mainly studied by means of simulation^23^^,^^25^^,^^86^^,^^94^ because it is difficult to isolate it in real experiments, whereas the skull effect was studied on a reduced number of subjects (N=10) and by NIRS simulations.^95^ Some more comprehensive studies focused on measurements of ischemic infarction patients, in which, however, averaged values for several cerebral positions were reported and limited information regarding the raw data/curves was provided.^77^^,^^96^

Despite the heterogeneous complexity of the morphology across the brain, the associated values partially indicate different brain conditions as well as depend on separate confounding factors. Anatomical lesions, disease progression, and therapeutic interventions (i.e., decompressive craniectomy) can all modify the regions beneath the probes. Nevertheless, TRS-DCS techniques offer some useful applications: i.e., the significant light absorption caused by hemorrhages can help diagnose injury types. In addition, the comparison of the optical properties of various areas of the head for patients where other stimulation is not possible by checking their symmetry, as previously reported, could be introduced. However, it is always preferable to refer to images of the underlying structures, if available, before performing any measurements.

Therefore, we believe that this work shows how the combination of hybrid DO with other techniques could enhance multimodal neuromonitoring, offering biomarkers that represent different aspects of pathogenetic mechanisms for a holistic (and more accurate) understanding of the brain status.

We provide a large, annotated dataset that could be used for further analysis and interpretation in the future.

It must be noted that the characterization reported in this paper, especially at the quantitative level, is affected by the properties and intrinsic features of the device used, which determine its performance and establish its limitations. Therefore, the experimental conditions and conclusions presented in this article should be reconsidered and adapted for each device/probe and cannot be fully generalized.

As previously stated, analyzing data with a model based on a homogeneous semi-infinite medium is not ideal in cases of heterogeneous probed volumes, such as for the data presented in this article. As noted in the literature reported in Secs 3.3, 3.2.3 and Table 4, a multi-layer analysis would be advantageous. However, to date, such models remain unstable due to the physics of the problem (strong dependence on specific assumed anatomical parameters) and have not been implemented as practical bedside, real-time analysis methods. Due to this, although preliminary exploratory analyses suggested potential differences in optical properties and blood flow among tissue types (e.g., skull bone and hematoma), these findings were not pursued further due to limitations in potential inaccuracies in quantitative values, as well as sample size and study design.

In this article, skin was not considered in the tissue assessment, because it was present in every measurement, even though the data are affected by its presence. In Ref. 23, the authors noticed that the light intensity was halved after scalp removal. Moreover, the scalp and skull possess highly diverse optical properties that provoke a completely different effect on the light passing through and the curve’s shape.^23^ The skin’s $\mu_{a}$ is quite higher than the skull’s: the authors argued that the skull would almost not attenuate the light passing through. Therefore, taking skin in relation to its thickness into account may be important.

A potential criticism is the choice to use only a few case examples from which to begin to discuss possible guidelines and improvements for using DO in these cerebral tissues. This approach might seem reductive, but the examples serve as a starting point. In a qualitative manner, we have considered various clinical and *in vivo* studies carried out in other populations such as ischemic infarction patients, patients with carotid stenosis, the elderly without any known brain injuries, and healthy subjects. Nonetheless, this study provides a good starting point for visualizing extreme cases of brain damage, though additional measurements are needed to reach solid conclusions.

Overall, this study provides case example studies of complex, yet common clinical situations, serving as a starting point for designing new devices, probes, and algorithms and for implementing quality control assessment. The output dataset can be utilized for further quantitative analysis, such as estimating optical properties for different tissue categories and carrying out numerical simulations. We strongly recommend that even real time, bedside display of the output should be supplemented with priors from the clinical and radiological evaluations to avoid false results. Finally, we highlight that there might be situations in which typical diffuse optical neuro-monitoring is simply impractical for certain tissue volumes.

## 5 Conclusions

In light of the findings and literature review, this article has successfully shown key morphological alterations and their relation to the performance of hybrid optical devices. The interpretations are truly multiple and yet necessary to confirm the practicality of this technique in the myriad of possible conditions.

## 6 Appendix A: Identification of the Regions of Interest on CT Scans

It was arbitrarily chosen to consider the ROI as a circular area with a radius of 15 mm and an area of $∼700\;{\mathrm{mm}}^{2}$. This choice was made upon the fact that the interfiber distance between the TRS source and detector tips for the device was 30 mm, and consequently, the optical sensor was probing with higher probability the density region at ∼15  mm deep.

Few steps were followed for the manual identification of each ROI. If the CT scan contained the reconstruction of the fiducial staples left on the patient’s skin (examples are provided in Fig. 2), the slice with higher contrast for this object was taken as an axial plane. The ROI was then selected “underlying” the staple contrast position. Unfortunately, there was no post-measurement CT scan with staples for all included subjects. In this eventuality, the retrieval of the ROI took advantage of the angular and radial information collected at the time of the acquisition. A mannequin head was used to relate distances and angles on a real volume and then adapt the position as much as possible to the morphology found in the CT.

For few subjects, the actual position was not recorded: in this case, an estimation based on the data collection experience was carried out. The procedure consisted of reaching the axial slice with the highest contrast for the crystalline biconvex lens of the eyes and then adding to this plane a vertical shift of 15 mm (the average value between the crystalline and the eyebrows for other subjects), adding further 40 mm to reach the middle of the forehead and select this axial plane. Then, in this plane, a line was depicted following the midline of the brain up to the front, and 45-deg angles were also drawn up to the forehead surface approximately in correspondence with the middle of the eyes and symmetrically for the two cerebral hemispheres. The crossing point on the forehead line was used to position the ROI in the area below, tangent to it.

## 7 Appendix B: CT Scans Example of Regular Versus Swollen Subcutaneous Tissue

**Fig. 14:**
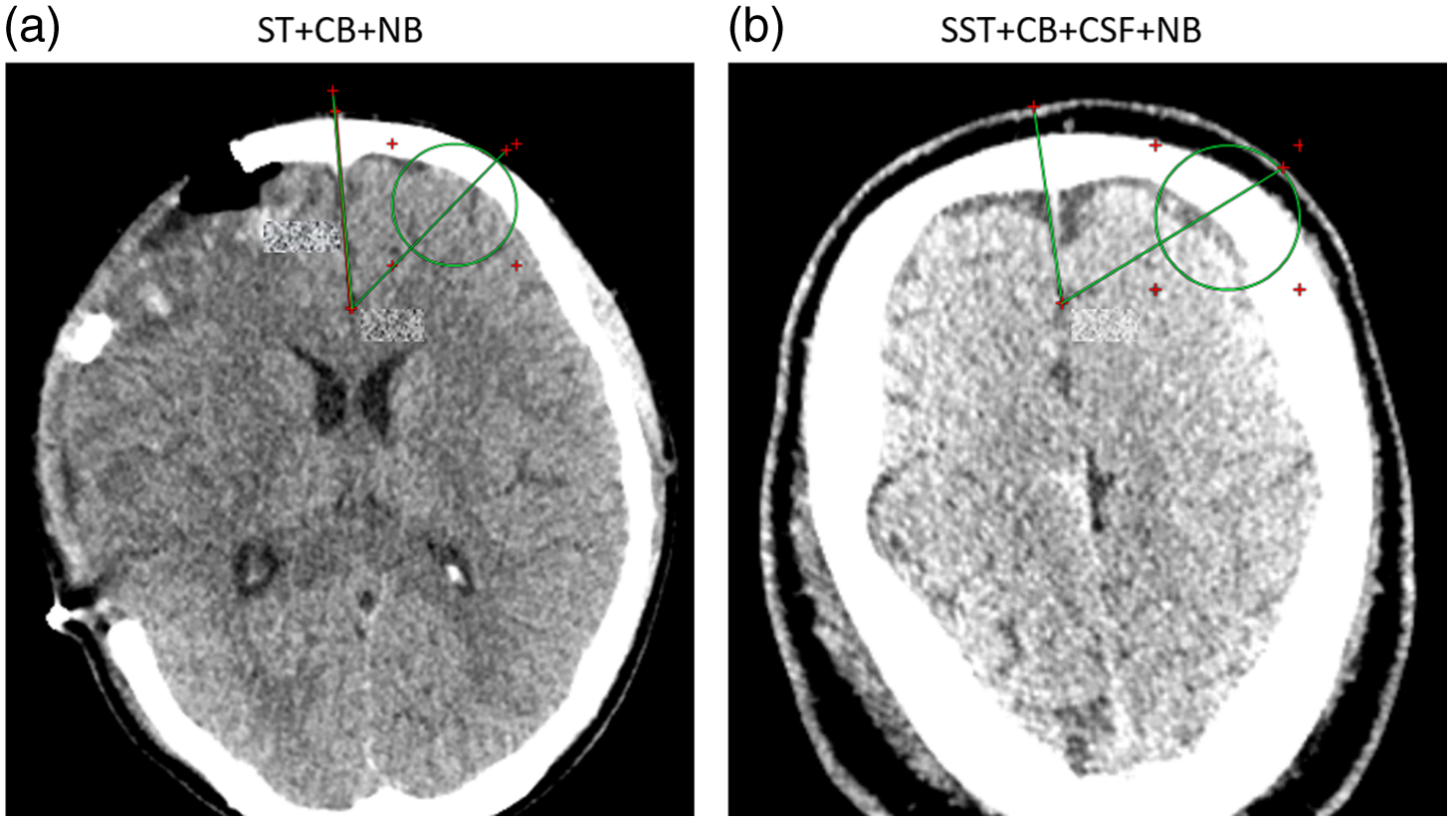
Examples of tissue composition encompassing regular ST and swollen tissue are provided to illustrate a reasonable understanding to inexperienced readers. (a) Green ROI was identified as having a tissue composition as “ST + CB + NB,” where no significant tissue thickness can be identified between skin and skull. (b) Instead, the green ROI circle includes a tissue labeled as “SST + CB + CSF + NB,” where there is a clear thick dark region between the skull (white) and the outer skin. Swollen tissue contains water, which explains the dark color in the CT scan for this region.

In Fig. 14, we facilitate a visual example of regular subcutaneous tissue in comparison with swollen subcutaneous tissue to make inexperienced users aware of the appearance of the CT scans. We would like to point out that expert personnel trained in interpreting CT scans were responsible for identifying the tissue categorization. Consequently, this task may not be as straightforward for inexperienced viewers to achieve.

## 8 Appendix C: **Expected Optical Signals Shape and Values Range**

We report the nature of typical signals measured from an uninjured, presumably healthy brain for those readers who are not familiar with TRS and DCS data.

Ideally, the decay for the DCS autocorrelation curve is exponential, decreasing with the square root of the delay time.^3^^,^^71^^,^^97^^–^^99^ When the normalization of the curve works as expected, the $g_{1}(\tau )$ curve starts near one and approaches exponentially to zero for increasing τ.^3^^,^^71^^,^^97^^–^^99^ For the source–detector separations and typical instrumentation, such as those used in this study, it reaches zero at $∼10^{-4}$ s.^3^^,^^71^^,^^97^^–^^99^ Even for a specific instrument, this is influenced by the BFI and other optical parameters.^3^^,^^37^ In a healthy brain, good-quality data are obtained with the observed count rate well above the noise level (0.5 kHz) and well below the saturation level (200 to 300 kHz, accounting for correlator and detector effects), with an instrument parameter (β) near 0.5 for unpolarized light.^3^^,^^13^^,^^100^^–^^102^ The fitted BFI value normal range is $1\times 10^{-9}$ to $8\times 10^{-8}\;{\mathrm{cm}}^{2}/s$^26^^,^^27^^,^^37^^,^^103^ which depends strongly on how the reduced scattering coefficient was calculated.

It is often the case that the DCS signal is affected by detector non-idealities such as dead time, shot noise, thermal noise, dark current, and afterpulsing probability, among others. As a side note, the afterpulsing effect of a detector is a phenomenon where, following a primary detection event, spurious pulses are generated that are not due to actual incident photons but are instead a result of the detector’s internal processes.^104^ This effect is particularly significant in some detector types, where residual charge carriers or trapped electrons from the initial detection event can be released at later times, causing additional false signals. For instance, the false afterpulsing pulses can create artificial correlations, leading to inflated values of the $g_{1}(\tau )$ at short delay times, a high β parameter, and cause additional noise.^105^ Although some of these could be “corrected” with a sufficiently thorough characterization of the individual detector behavior, it is not advisable to do so when these become the dominant processes due to low photon counts.

In an ideal scenario, the “TRS curves” from both wavelengths show an expected behavior: the peak of the DTOF is shifted to later times with respect to the IRF peak, and the curve is broader meaning that the FWHM of the DTOF is much larger than that of the IRF due to the broad distribution of the propagation times in tissue.^33^^,^^62^ DTOFs are generally attenuated in amplitude (often visualized and/or processed normalized to the peak) with a long decaying tail. This tail decays linearly for long path lengths where absorption occurs with greater probability and dominates over scattering.^33^^,^^62^ The delay of the peak comes from the finite time that light employs to go from the source to the detector.^33^ The broadening of the DTOF is a consequence of the different paths that photons travel inside of media because of multiple scattering events.^33^^,^^62^

Retrieving the proper shape of the DTOF, built by the number of detected photons yields information about the optical properties of scattering and absorption that characterize the probed medium area. To obtain a good quality signal, the maximum signal level has to be a few decades above the noise background prior to the DTOFs.^33^^,^^62^ Moreover, both DTOFs at the two wavelengths should behave similarly, which is expected in a healthy-like tissue where oxy- and deoxyhemoglobins affect them in a similar way. This could change dramatically in case of deeply deoxygenated, i.e., often hypoxic and/or ischemic, tissue. Overall, a dynamic range of three to four decades (or higher) for the DTOF curves is generally acceptable to ensure a proper fit of the optical parameters, especially guaranteeing to extrapolate the absorption information from the linear decay of the tail.^62^ The DTOF peak delay from the IRF peak of at least ∼0.7 to 0.8 ns is also common and is generally acceptable;^33^ otherwise, it may entail insufficient diffusion time of the photons within the medium, hence rendering accurate the measurement of IRF even more crucial.

The relative FWHM is expected to be a value greater than one when there is typical broadening due to photon diffusion in the tissue. In fact, the FWHM of the IRF defines the temporal resolution of the device. When the photon’s arrival time broadens due to the scattering events and the emerging DTOF modifies its decay given the amount of absorption events while traversing a tissue, the output DTOF retains this information: the more scattering events (and properties, the more), the broader the histogram of photon arrival times. Therefore, the FWHM of the DTOF is generally broader than the IRF one.^62^ This is typical for a structure such as the healthy human head and its optical properties.^58^

ΔFWHM should be positive in standard diffusion/absorption conditions.^40^^,^^106^

The SNR level depends on many factors, such as the background noise of the detectors or how well external light is shielded. Its acceptable value has to guarantee a clear distinction between the DTOF curve and the background level,^106^ and generally, an SNR = 10 can be chosen as a non-strict value below which data should be considered for exclusion. Generally, a rule of thumb for good values in a healthy brain are $\mu_{a}∼{\mathrm{cm}}^{-1}$, $\mu_{s}^{\prime }∼10\;{\mathrm{cm}}^{-1}$, and ${\mathrm{StO}}_{2}$ around 56% to 66%.^7^^,^^33^^,^^58^ Nonetheless, these are indicative values and literature examples carried out on large cohorts of patients are useful as a starting point, i.e., from Refs. 58, 77, and 88.

In both cases, we have provided a qualitative guideline based on experience and the literature that could be adopted, even at the bedside as a figure of merit. For further quantitative analysis, we refer the readers to excellent reviews of the topics such as in Refs. 32, 33, 58, 69, 88, 107, and 108 and their citations.

## 9 Appendix D: Additional Healthy Case Examples

**Fig. 15:**
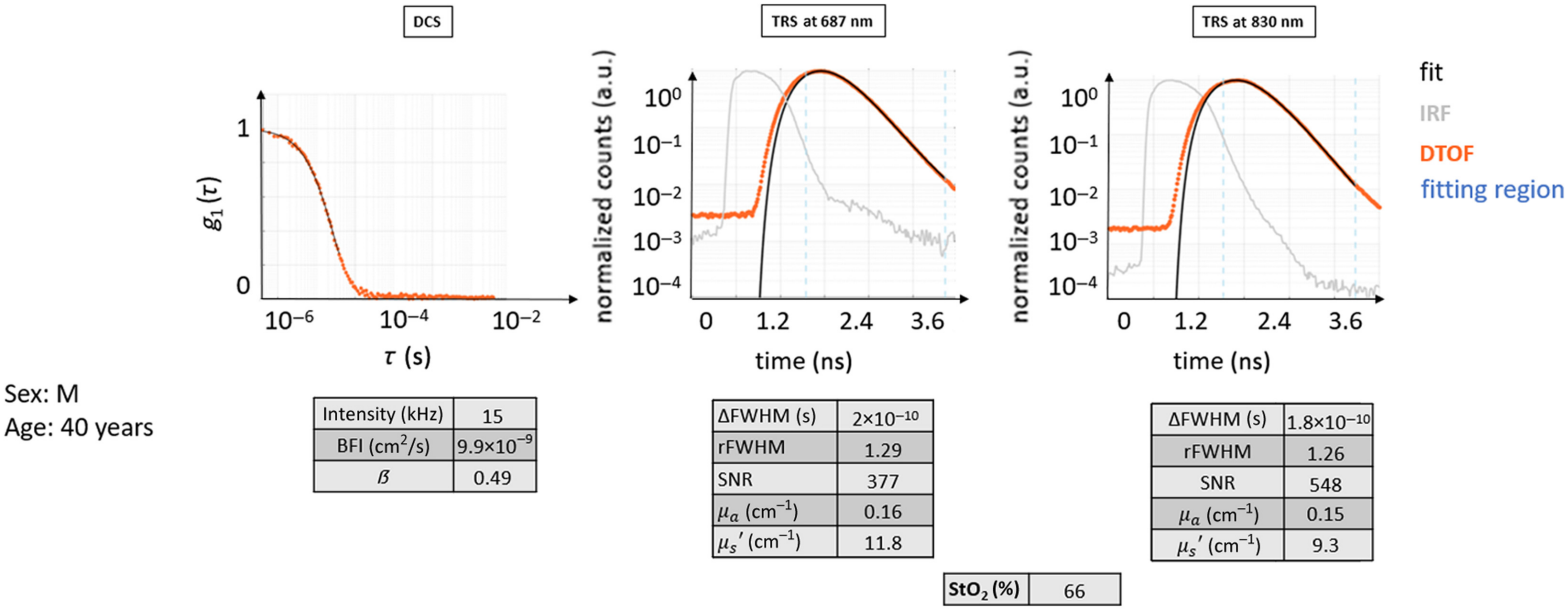
Typical curves for TRS and DCS measured on a healthy volunteer: healthy case H1. The DTOF curves and their fit are shown. The fitting region of the DTOF curves is indicated in light blue dashed lines. It is important to point out that in the DTOF graphs, the 0 ns on the time axis was arbitrarily set and does not coincide with the peak of the IRF curve to be able to show the entire curve and its initial background. The typical distance between the IRF curves and their diffuse DTOFs can be appreciated, as well as the slower linear decay of the tail of the DTOF for both wavelengths. The latter also presents almost a decade of higher background before the curve, with a consequently decreased dynamic range for the DTOF. The fit follows very closely the curves of raw data points. The autocorrelation curve also presents a typical behavior as described in the text.

**Fig. 16:**
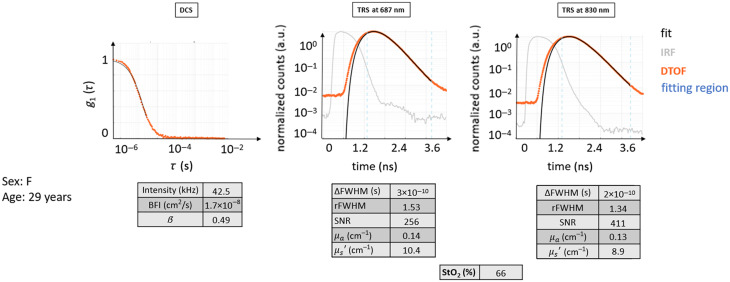
Typical curves for TRS and DCS measured on a healthy volunteer: healthy case H2. The DTOF curves and their fit are shown. The fitting region of the DTOF curves is indicated in light blue dashed lines. It is important to point out that in the DTOF graphs, the 0 ns on the time axis was arbitrarily set and does not coincide with the peak of the IRF curve to be able to show the entire curve and its initial background. The typical distance between the IRF curves and their diffuse DTOFs can be appreciated, as well as the slower linear decay of the tail of the DTOF for both wavelengths. The latter also presents almost a decade of higher background before the curve, with a consequently decreased dynamic range for the DTOF. The fit follows very closely the curves of raw data points. The autocorrelation curve also presents a typical behavior as described in the text.

**Fig. 17:**
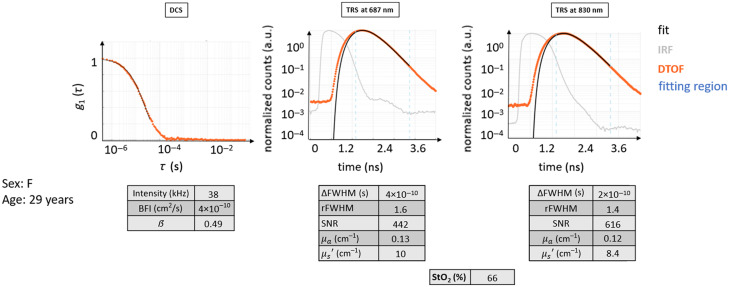
Typical curves for TRS and DCS measured on a healthy volunteer: healthy case H3. The DTOF curves and their fit are shown. The fitting region of the DTOF curves is indicated in light blue dashed lines. It is important to point out that in the DTOF graphs, the 0 ns on the time axis was arbitrarily set and does not coincide with the peak of the IRF curve to be able to show the entire curve and its initial background. The typical distance between the IRF curves and their diffuse DTOFs can be appreciated, as well as the slower linear decay of the tail of the DTOF for both wavelengths. The latter also presents almost a decade of higher background before the curve, with a consequently decreased dynamic range for the DTOF. The fit follows very closely the curves of raw data points. The autocorrelation curve also presents a typical behavior as described in the text.

**Fig. 18:**
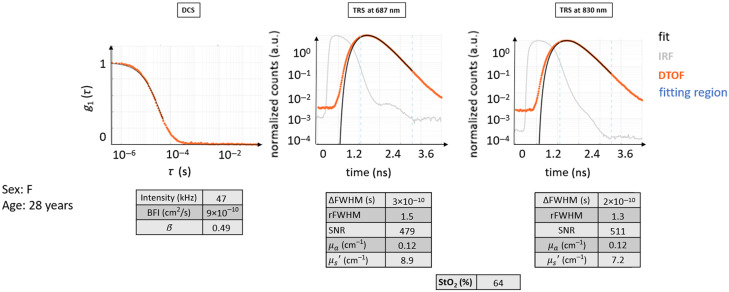
Typical curves for TRS and DCS measured on a healthy volunteer: healthy case H4. The DTOF curves and their fit are shown. The fitting region of the DTOF curves is indicated in light blue dashed lines. It is important to point out that in the DTOF graphs, the 0 ns on the time axis was arbitrarily set and does not coincide with the peak of the IRF curve to be able to show the entire curve and its initial background. The typical distance between the IRF curves and their diffuse DTOFs can be appreciated, as well as the slower linear decay of the tail of the DTOF for both wavelengths. The latter also presents almost a decade of higher background before the curve, with a consequently decreased dynamic range for the DTOF. The fit follows very closely the curves of raw data points. The autocorrelation curve also presents a typical behavior as described in the text.

In this appendix, we provide further examples of typical TRS and DCS curves from healthy subjects to not only provide further data as an illustration but also to demonstrate that even the presumably healthy brain impresses significant heterogeneity on the measurement.^32^^,^^33^^,^^57^^,^^58^^,^^109^ We have produced them in the same fashion as healthy case H0 in Figs. 15–18.

## 10 Appendix E: Summary of the Case Examples

The case examples are summarized in Table 5 to facilitate the comparison among cases.

**Table 5:** Summary of the case examples presented in the results, to help the reader in their comparison. TBI, traumatic brain injury; SAH, subarachnoid hemorrhage; MCA, malignant cerebral artery stroke; SNR, signal-to-noise ratio; BFI, blood flow index; $\mu_{a}$, absorption coefficient; $\mu_{s}^{\prime }$, reduced scattering coefficient; CSF, cerebrospinal fluid.

Case	Cohort	Tissue composition	Physiology	TRS		DCS	
				Finding	Conclusion	Finding	Conclusion
1	MCA	ST + EH + IT	Hemi-craniectomy and infarct	Unacceptably poor SNR at 687 nm and low intensity at 830 nm	Large amounts of highly deoxygenated blood causing significant absorption	High signal intensity but very low BFI	Very low BFI in the tissue
2	TBI	SST + CSF + CB/A	Diffuse axonal injury with epidural hematoma (left) and cranial fractures followed by craniotomy	High $\mu_{a}$ and $\mu_{s}^{\prime }$ at both wavelengths	Direct light affecting the signal or low scattering due to underlying swollen muscle (liquid)	High BFI with fast decay to 0 and low light intensity	Low light reaches the tissue due to low scattering and high absorption and fast decay due to high absorption
3	TBI	SST + CSF + NB	Subdural hematoma and subarachnoid hemorrhage with decompressive craniectomy leading to swollen muscle with large edema	Fast decay but sufficient amplitude and high $\mu_{a}$ but low $\mu_{s}^{\prime }$	Direct light may have affected the signal or low scattering due to underlying swollen muscle filled with liquid	Slow decay and BFI lower than normal	The absence of a skull may account for the finding
4	TBI	SST + CB + CSF + NB	Ischemic lesion on the capsule–thalamic region and frontoparietal hemorrhage on the left, treated with craniotomy (right)	Low intensity, especially at 687 nm, and low $\mu_{s}^{\prime }$	High deoxygenated level affecting mainly at 687 nm and reduced scattering	Unusable DCS curve affected by noise	Signal intensity probably absorbed by hair follicles and lower backscattered light due to the CSF
5	TBI and SAH	ST + CB + ICH/NB	Diffuse lesion type III with decompressive craniectomy on the left	Normal $\mu_{a}$, but high $\mu_{s}^{\prime }$ and ${\mathrm{StO}}_{2}$	Low oxygen extraction typical of hemorrhagic tissue	Low intensity, high β, damped shape, and no single exponential	Unreliable BFI due to improper fitting and hemorrhage may affect $g_{1}(\tau )$ shape
6	TBI and SAH	ST + CB + SAH + NB	Subdural hemorrhage following a concussion on the left necessitating a craniotomy to extract the hematoma	Fast linear decay, high ${\mathrm{StO}}_{2}$, and low $\mu_{s}^{\prime }$	Impossibility to discard data qualitatively and values suggest altered morphology	Final portion of $g_{1}(\tau )$ does not reach 0, secondary exponential decay, significant deviation from fit, and unreliable BFI	Sudden change in morphology causing atypical tissue and shallow region with low BFI
7	TBI	ST + CB + CSF + NB	Ischemic lesion on the capsule-thalamic region and frontoparietal hemorrhage on the left, treated with craniotomy (right)	Similarity to healthy-like and low ${\mathrm{StO}}_{2}$	Impossibility to qualitative distinguish from normal tissue, CSF presence difficult to recognize, low ${\mathrm{StO}}_{2}$ probably due to tissue thickness above the brain, and unlikely reached	Distorted $g_{1}(\tau )$ with multiple decay constants and unreliable fit: no BFI	Change in exponential decay symptomatic of layered tissue
8	TBI	SST + CB + A + NB	Concussion followed by craniectomy (left)	Healthy-like curves and high ${\mathrm{StO}}_{2}$ and $\mu_{s}^{\prime }$	Impossibility to qualitatively distinguish from normal and air leads to complex results confusing traditional quality control methods	Secondary decay at the end of $g_{1}(\tau )$, low intensity, and values similar to normal	Air leads to complex results confusing traditional quality control methods

## Data Availability

Data are available on CORA, Repositori de dades de Recerca repository at https://doi.org/10.34810/data1355, under the title *Replication Data for: How the heterogeneity of the severely injured brain affects hybrid diffuse optical signals: case examples and guidelines*. The CT scans can be downloaded upon request to the authors.
